# Effects of Iron Oxide Nanoparticles (γ-Fe_2_O_3_) on Liver, Lung and Brain Proteomes following Sub-Acute Intranasal Exposure: A New Toxicological Assessment in Rat Model Using iTRAQ-Based Quantitative Proteomics

**DOI:** 10.3390/ijms20205186

**Published:** 2019-10-19

**Authors:** Dalel Askri, Valérie Cunin, Souhir Ouni, David Béal, Walid Rachidi, Mohsen Sakly, Salem Amara, Sylvia G. Lehmann, Michel Sève

**Affiliations:** 1 University Grenoble Alpes, PROMETHEE Proteomic Platform, BEeSy, 38000 Grenoble, France; VCunin@chu-grenoble.fr (V.C.); sylviaglehmann@outlook.fr (S.G.L.); Michel.Seve@univ-grenoble-alpes.fr (M.S.); 2 LBFA Inserm U1055, PROMETHEE Proteomic Platform, 38000 Grenoble, France; 3 Institut de Biologie et Pathologie, PROMETHEE Proteomic Platform, CHU Grenoble Alpes, 38000 Grenoble, France; 4 Carthage University, College of Sciences of Bizerte, Unit of Research in Integrated Physiology, Bizerte 7021, Tunisia; souhir.ouni.chaouali@gmail.com (S.O.); mohsensakly@gmail.com (M.S.); amara_salem_fsb@yahoo.fr (S.A.); 5 University Grenoble Alpes, SyMMES/CIBEST UMR 5819 UGA-CNRS-CEA, INAC/CEA-Grenoble LAN, 38000 Grenoble, France; david.beal@cea.fr (D.B.); walid.rachidi@univ-grenoble-alpes.fr (W.R.); 6 Shaqra University, Faculty of Sciences and Humanities, Department of Natural and Applied Sciences in Afif, Afif 11921, Saudi Arabia; 7 University Grenoble Alpes, University Savoie Mont Blanc, CNRS, IRD, IFSTTAR, ISTerre, 38000 Grenoble, France

**Keywords:** iron oxide nanoparticles, proteomics, in vivo, toxicity, rat

## Abstract

Iron Oxide Nanoparticles (IONPs) present unique properties making them one of the most used NPs in the biomedical field. Nevertheless, for many years, growing production and use of IONPs are associated with risks that can affect human and the environment. Thus, it is essential to study the effects of these nanoparticles to better understand their mechanism of action and the molecular perturbations induced in the organism. In the present study, we investigated the toxicological effects of IONPs (γ-Fe_2_O_3_) on liver, lung and brain proteomes in Wistar rats. Exposed rats received IONP solution during 7 consecutive days by intranasal instillation at a dose of 10 mg/kg body weight. An iTRAQ-based quantitative proteomics was used to study proteomic variations at the level of the three organs. Using this proteomic approach, we identified 1565; 1135 and 1161 proteins respectively in the brain, liver and lung. Amon them, we quantified 1541; 1125 and 1128 proteins respectively in the brain, liver and lung. Several proteins were dysregulated comparing treated samples to controls, particularly, proteins involved in cytoskeleton remodeling, cellular metabolism, immune system stimulation, inflammation process, response to oxidative stress, angiogenesis, and neurodegenerative diseases.

## 1. Introduction

Humans are exposed to various nanoscale materials on a daily basis, particularly after the development of nanotechnology, posing a threat to human life and the environment. Due to their small size, NPs have the ability to easily penetrate the human body, to cross the various biological barriers and to reach the most sensitive organs [1]. Some NPs may induce toxicity that should be known and studied to fully understand the mode of action of these NPs in the body. Since last decades, researches based on the toxicity of nanomaterials have intensely been conducted by scientific researchers worldwide. Indeed, nanomaterials such as nanoparticles, nanotubes or nanospheres are used in several domains especially in electronics, cosmetics, medicine and biotechnology [2,3,4,5]. Several studies reported the importance and the value added by these kinds of materials in our life. Nevertheless, considered number of publications put the accent on the disadvantages of the nanomaterials and their risk and dangerousness for the Human, animals and environment. Thus, several in vitro and in vivo studies have been conducted in this setting, using several cellular models (endothelial cells, macrophages, cancer cells) [6,7,8,9,10] and animal models (rat, mice, Zebrafish) [11,12,13,14,15,16]. In rats, different routes of exposure such dermal, intravenous, intranasal and oral were used. The results obtained depend in particular on the size of the NPs, the route of administration, the dose, and the duration of the exposure. NPs are known to cause different biological responses, including generation of reactive oxygen species (ROS) [17], modification of cell signaling, as well as stimulation of increased expression of pro-inflammatory cytokines without causing cytotoxicity [18]. IONPs have been demonstrated to induce necrosis through ROS production on an A549 lung adenocarcinoma line and on normal fibroblasts [19] and to be cytotoxic and genotoxic by the induction of oxidative DNA damage and apoptosis on an MCF-7 breast cancer cell line [20]. Wang et al. (2010) reported the toxic effects of Fe_2_O_3_ NPs inhalation on Wistar rats that cause significant damage to the liver and lungs [21]. Sadeghi et al. (2015) [22] have shown that the accumulation of IONPs leads to histopathological toxicity in the liver and lungs. Similarly, oxidative stress was studied in this work, the team showed an increase in the production of dose-dependent ROS, with a decrease in glutathione (GSH) levels which represents one of the most important endogenous barriers against this stress. This proves the weakness of the antioxidant barrier against these iron NPs. It is known that NPs could be accumulated by the body. The major organs where the NPs are likely to end-up depend on the route of administration and are mainly the liver, the spleen and the lung [23]. The study of the toxicity of nanoparticles or nanomaterials in general was not limited to classic toxicological studies. Interestingly, over the last few years, a new kind of studies has emerged namely the nanotoxicoproteomics [24,25]. This new field means the use of proteomics to investigate the toxicity of nanomaterials. Indeed, using this approach, whole proteomes could be examined to visualize unexpected responses to NPs. Nanotoxicoproteomics as a sophisticated approach coupled with advanced bioinformatic analysis has allowed the identification and quantification of altered proteins that could provide a new avenue for biomarker discovery due to NP exposure [26]. In fact, the use of proteomic techniques to identify potential biomarkers linked to nanomaterial exposure could be a powerful tool to evaluate the risks and to prevent diseases associated with the nano exposure. To explain more, the discovery of the proteome changes linked to a hazardous exposure to NPs could help to get deep insights into exposure effects and the toxicity mechanisms of the NPs in which various proteins play a major role. In order to study the effects of NPs on the proteome and to decipher the mechanisms of their potential toxic effects, various nanotoxicoproteomic studies have been conducted in vitro and in vivo using different NPs such as Au-NPs, Ag-NPs, CuO-NPs, SiO2-NPs, TiO2-NPs, ZnO-NPs and Fe-NPs [27]. Meanwhile, no studies have been conducted to evaluate the effects of IONPs, γ-Fe_2_O_3_, on rat and especially on the organs such as brain, liver and lung. Therefore, in the present study, we aimed to investigate the proteomic profiling data of IONP sub-acute exposure through the intranasal route in specific organs namely the brain, the liver and the lung.

## 2. Results and Discussion

The proteomic study based on iTRAQ isotope labeling followed by OFF-Gel fractionation and MS/MS mass spectrometry is a good strategy for studying the effects of the NPs that are in our study iron NPs. In recent years, several studies have been conducted to investigate the mechanisms of action of NPs and the biological processes that they could disrupt at the cellular level using proteomic tools in in vitro or in vivo studies [24,27].

In order to study the molecular mechanisms by which nanoparticles can act, we carried out a proteomic study “Bottom-up” based on mass spectrometry and iTRAQ isotope labeling followed by OFFGEL fractionation as previously described by Lehmann et al. 2017; 2019 and Askri et al. 2019 [28,29,30]. The lung, liver and brain were chosen to study their respective proteomes following intranasal exposure to iron NPs for 7 consecutive days at a daily dose of 10 mg/kg. Indeed, as reported by Askri et al. 2018 [31] and after an intranasal exposure to IONPs, we observed interesting effects on the biochemical parameters of the rats. Thus, we decided to analyze the protein variations at the level of the liver. Furthermore, the intranasal instillation is directly associated and connected to the nervous and respiratory systems. Indeed, it is well known that the NPs can be translocated to the brain through the olfactory nerve and translocated to the lungs by the respiratory tract having as a gateway the nose [32,33]. Thus, we chose to study the proteomes of the brain and the lung next to the proteome of the liver.

iTRAQ isotope labeling is well suited to protein biomarkers and drug discovery studies linked to a specific disease or profile. It allows both identification and quantification of proteins in an efficient multiplexing of samples, by highlighting the differentially expressed proteins (DEP) of each sample corresponding to each condition. The peptides resulting from trypsin digestion have been labeled with the iTRAQ reagents, which will make it possible to quantify the proteins that vary in response to IONP exposure. Thus, the ratios between organs from treated and control rats indicate the relative abundance of proteins under the different conditions.

In this research work, using our non-targeted proteomic screening and Isobar Package analysis, we have identified 1565 proteins and quantified 1541 proteins in the brain, identified 1135 proteins and quantified 1125 proteins in the liver and finally, identified 1161 proteins and quantified 1128 proteins in the lung. Of these, 127 were significantly differentially expressed (8%) between treated and control rats at the cerebral level with 67 down-regulated and 60 up-regulated (Table S1), 66 DEP (6%) at the liver level with 35 down-regulated and 31 up-regulated (Table S2) and 84 DEP (8%) in the lungs with 30 down-regulated and 54 up-regulated (Table S3). This suggests that the dose of 10 mg/mL used for 7 consecutive days was well chosen to induce small variations in the proteomes studied without inducing the death of the animal.

DEPs identified and quantified, beforehand, at each organ level were analyzed using bioinformatic tools. The down or up-regulated proteins were analyzed by the TargetMine [34], PANTHER [35] and STRING [36] softwares to study the biological pathways, the processes in which they are involved and the Protein-Protein interactions.

**Figure 1:**
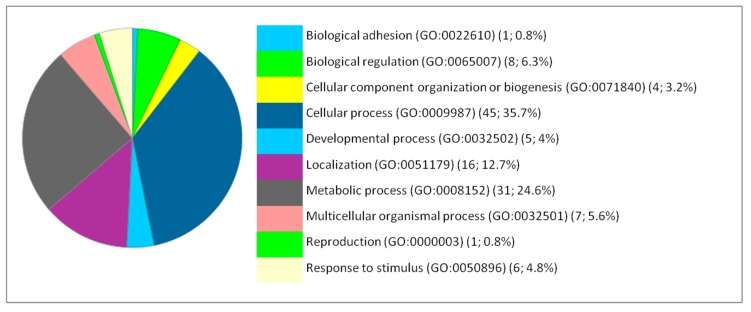
Functional classification of the 127 differentially expressed proteins in the brain following IONP exposure according to the biological processes. The pie chart was generated from PANTHER Classification System. The number and percentage are given based on the 127 proteins. Biological process name (GO: Gene Ontology class ID) (number of proteins; percentage from 127 proteins).

**Figure 2:**
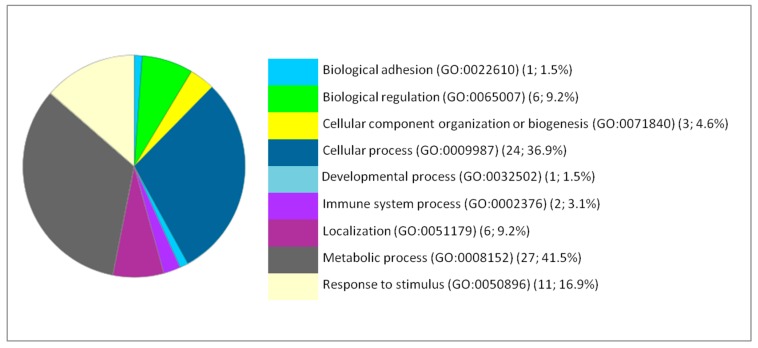
Functional classification of the 66 differentially expressed proteins in the liver following IONP exposure according to the biological processes. The pie chart was generated from PANTHER Classification System. The number and percentage are given based on the 66 proteins. Biological process name (GO: Gene Ontology class ID) (number of proteins; percentage from 66 proteins).

**Figure 3:**
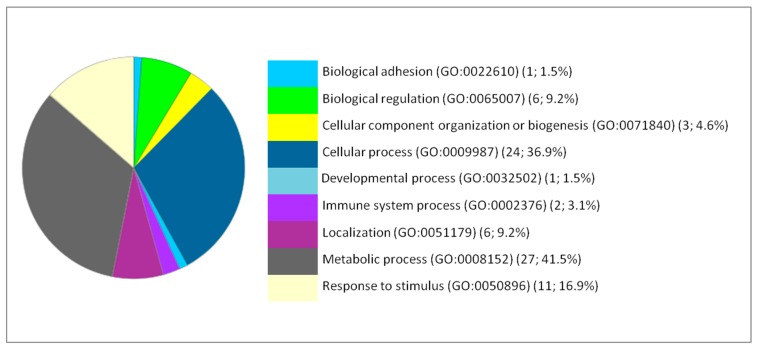
Functional classification of the 84 differentially expressed proteins in the lung following IONP exposure according to the biological processes. The pie chart was generated from PANTHER Classification System. The number and percentage are given based on the 84 proteins. Biological process name (GO: Gene Ontology class ID) (number of proteins; percentage from 84 proteins).

The impact of iron NPs on the various pathways related to brain activity and function suggests potential toxic effects of these NPs that may be more likely to manifest clearly with duration of exposure or with increasing dose. The effect of NPs on hepatic function stimulated the anti-oxidative stress response. Likewise, the response was also metabolic because the most remarkable effects were on the metabolic pathways such as the glycolysis pathway, the pentose phosphate pathway, the pyruvate metabolism and the synthesis of ATP and amino acids (arginine, glycine and serine). Based on the Gene Ontology analysis, the DEP proteins are involved in various biological processes. The main affected biological processes by iron NPs exposure have been identified by gene ontology using PANTHER and are illustrated in Figure 1, Figure 2 and Figure 3.

The functional classification of the biological processes in which the DEP are involved at the levels of the brain, liver and lung showed a few similarities between the cellular responses of the different organs. Thus, the most affected process is the metabolism for the liver. Therefore, we focused our analysis on the metabolic processes to have a second level of investigation. In the liver, the metabolic processes present 41.5% from the corresponding processes to the 66 DEP proteins with 27 proteins. The metabolic processes are detailed as follows: Biosynthetic process (GO:0009058) (2; 7.4%), Catabolic process (GO:0009056) (6; 22.2%), Cellular metabolic process (GO:0044237) (15; 65.6%), Organic substance metabolic process (GO:0071704) (18; 66.7%), Oxidation-reduction process (GO:0055114) (2; 7.4%), Primary metabolic process (GO:0044238) (6; 22.2%), and Small molecule metabolic process (GO:0044281) (5; 18.5%).

The metabolism is in the second position for the brain and the lung. In the brain, the metabolic processes present 24.6% from the corresponding processes to the 127 DEP proteins with 31 proteins. The metabolic processes are detailed as follows: Biosynthetic process (GO:0009058) (2; 6.5%), Catabolic process (GO:0009056) (4; 12.9%), Cellular metabolic process (GO:0044237) (14; 45.2%), Organic substance metabolic process (GO:0071704) (25; 80.6%), Oxidation-reduction process (GO:0055114) (4; 12.9%), Pigment metabolic process (GO:0042440) (1; 3.2%), Primary metabolic process (GO:0044238) (4; 12.9%), and Small molecule metabolic process (GO:0044281) (2; 6.5%). In the lung, the metabolic processes present 41.5% from the corresponding processes to the 66 DEP proteins with 27 proteins. The metabolic processes are detailed as follows: Biosynthetic process (GO:0009058) (4; 20%), Catabolic process (GO:0009056) (2; 10%), Cellular metabolic process (GO:0044237) (13; 65%), Hormone metabolic process (GO:0042445) (1; 5%), Organic substance metabolic process (GO:0071704) (12; 60%), Oxidation-reduction process (GO:0055114) (2; 10%), Pigment metabolic process (GO:0042440) (1; 5%), Primary metabolic process (GO:0044238) (3; 15%), Small molecule metabolic process (GO:0044281) (4; 20%).

**Figure 4:**
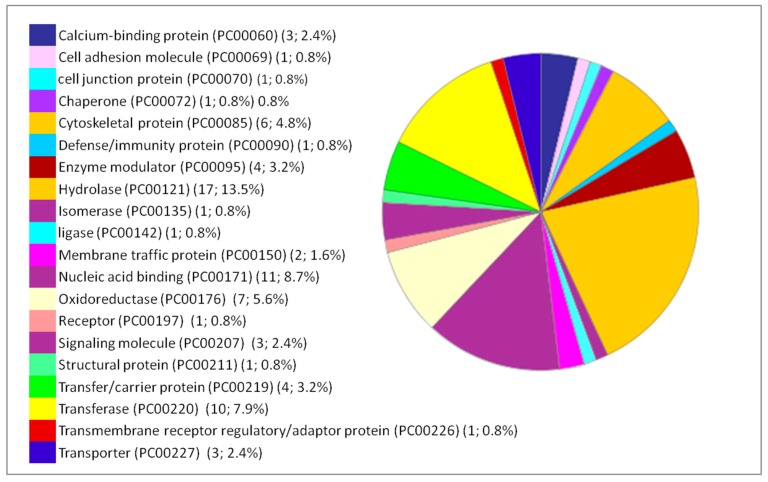
Functional classification of the 127 differentially expressed proteins in the brain following IONP exposure according to PANTHER protein class. The pie chart was generated from PANTHER Classification System. The number and percentage are given based on the 127 proteins. Protein name (Protein class PC) (Number of proteins; percentage from the 127 proteins).

**Figure 5:**
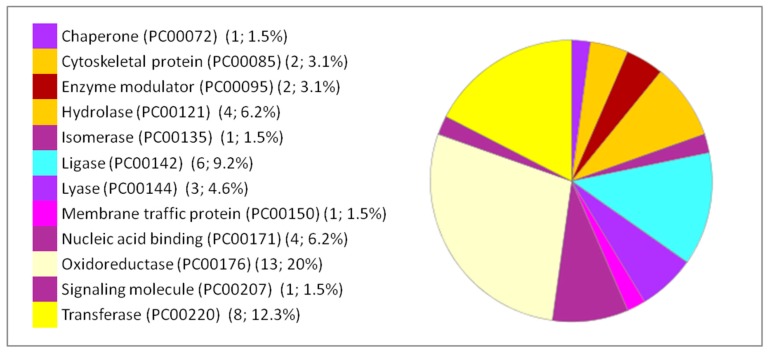
Functional classification of the 66 differentially expressed proteins in the liver following IONP exposure according to PANTHER protein class. The pie chart was generated from PANTHER Classification System. The number and percentage are given based on the 66 proteins. Protein name (Protein class PC) (Number of proteins; percentage from the 66 proteins).

**Figure 6:**
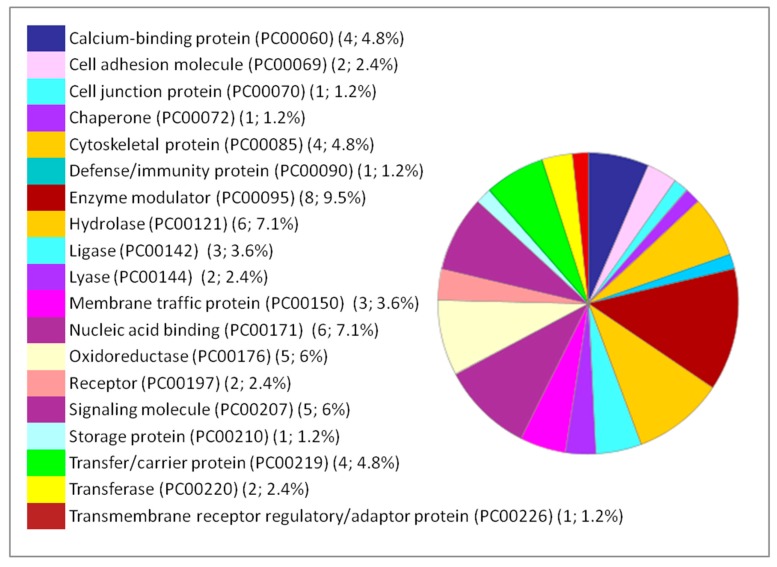
Functional classification of the 84 DEPs in the lung following IONP exposure according to PANTHER protein class. The pie chart was generated from PANTHER Classification System. The number and percentage are given based on the 84 proteins. Protein name (Protein class PC) (Number of proteins; percentage from the 84 proteins).

Moreover, the percentages of other processes did not show considerable differences among the three organs studied, such as cellular processes, cellular organization, biological regulation, and response to stimuli. The immunological response is minimal compared to the biological processes mentioned above but it appears only in the liver and lung. Classes of the dysregulated proteins following IONP exposure have been also studied by PANTHER. The results showed diverse classes that are related to the previous mentioned biological processes affected in response to the exposure to the NPs. Common classes were identified between the dysregulated proteins in each of the organs (brain, liver and lung). The results are presented in Figure 4, Figure 5 and Figure 6.

PANTHER analysis of the liver proteome has shown that dysregulated proteins are mostly involved in various pathways as detailed in Table 1.

The analysis of the cerebral proteome has shown that dysregulated proteins are involved in various signaling pathways mainly related to the nervous system such as the pathways indicated in Table 2. Overexpressed proteins are also involved in the signaling pathways of inflammatory, immune, apoptotic and cancerous processes (Table 3).

The lung proteins are involved in several signaling pathways combining those found in the brain and the liver. Between those pathways we could cite: Integrin signaling pathway (P00034), Metabotropic glutamate receptor group I pathway (P00041), B cell activation (P00010), Metabotropic glutamate receptor group II pathway (P00040), Glutamine glutamate conversion (P02745), Beta2 adrenergic receptor signaling pathway (P04378), Huntington disease (P00029), Beta1 adrenergic receptor signaling pathway (P04377), Heterotrimeric G-protein signaling pathway-rod outer segment phototransduction (P00028), Pyrimidine Metabolism (P02771), Heterotrimeric G-protein signaling pathway-Gq alpha and Go alpha mediated pathway (P00027), Heterotrimeric, G-protein signaling pathway-Gi alpha and Gs alpha mediated pathway (P00026), 5HT1 type receptor mediated signaling pathway (P04373), Glycolysis (P00024), GABA-B receptor II signaling (P05731), T cell activation (P00053), Arginine biosynthesis (P02728), and Plasminogen activating cascade (P00050).

To go deeper in our analysis regarding the nanoparticle effects, we focused on biological processes that are related to the known responses to NPs in general at the level of each organ. The analysis of proteins and processes was done with the free software “TargetMine” and the results are expressed in the Table 4, Table 5 and Table 6 for proteomes of the brain, liver and lung successively. At the brain level (Table 4), interesting proteins were found to be over-expressed following IONP exposure. The main corresponding biological processes are the response to oxidative stress, Inflammation, carcinogenesis and neuronal damage. However, the under-expressed proteins are involved in adherence, neuronal differentiation and signal transduction. This may reflect the potential neuronal toxic effects of IONP on the brain.

Interestingly, at the hepatic level, the upregulated proteins are involved in biological processes known as the “Nano” response such as the response to oxidative stress and the stimulation of the immune system. Via the blood, iron NPs reach the liver and induce local oxidative reactions. The body tends to defend itself, which inevitably leads to an increase in antioxidant biomarkers. However, the under-expressed proteins are mostly involved in the metabolism. The table below (Table 5) summarizes the main liver proteins over-expressed or under-expressed following the intranasal administration of NPs.

In the lung, overexpressed proteins are involved in oxidative stress responses, immune system stimulation and inflammation response. These processes are known to be involved in the responses to NPs as it is the case for copper [37,38] and silver [39] NPs. The effect at the pulmonary level seems to be more intense than at the hepatic level. This can be explained by the fact that iron NPs were administered through the intranasal route. IONPs can translocate directly via the bloodstream to the lungs and induce potentially toxic effects hence stimulating the antioxidant and anti-inflammatory response. Down-regulated proteins in the lung are involved in metabolism, oxygen transport, hemostasis and cell differentiation and proliferation. Examples of those proteins are summarized in Table 6.

The analysis of the different signaling pathways and biological processes has shown that the proteomes of the studied brain, liver and lung organs have similarities and divergences in the way of interacting towards the iron NPs. In fact, following the intranasal administration of iron NPs, the brain seems to be impacted more or less equally to that of the lungs. This observation could be quite correct because the intranasal route is directly linked to the brain by the olfactory nerve and to the lungs by the respiratory system. Compared to our previous in vitro study [30], the present proteome analysis of the brain, liver and lung showed a destabilization of the cytoskeleton mainly in the brain. The following table (Table 7) illustrates the different deregulated proteins that are involved in cytoskeletal remodeling.

Interestingly, none of these proteins is common between two or three organs proving the specificity of deregulated proteins. A complementary analysis of the Protein-protein interactions (PPI) using STRING Database (Version 11.0) showed that for each organ, several proteins were significantly connected to each other (Figures S1–S3). The cerebral proteins showed more connections and stronger network than the lung and the liver with 125 nodes and 183 edges. The PPI network evidenced 3 clusters: cluster 1 containing the ribosomal proteins such as 60S ribosomal protein L7, ribosomal protein S27a, Ribosomal protein L18A, Ribosomal protein S15 and 60S acidic ribosomal protein P2. The second cluster contains the NADH dehydrogenases namely: NADH dehydrogenase (ubiquinone) 1 beta subcomplex, 7, NADH dehydrogenase [ubiquinone] iron-sulfur protein 4, NADH dehydrogenase (ubiquinone) Fe-S protein 8, NADH: ubiquinone oxidoreductase subunit B4 The third cluster contains proteins from the Ras superfamily of small G proteins: Ras-related protein Rab-2A, Rab-3C, Rab-14 and Rab-11A. The hepatic proteins presented a protein network of 61 nodes and 103 edges. The network evidenced several metabolic enzymes or proteins linked to each other such as the ATP-citrate synthase, Fatty acid synthase, Glucose-6-phosphate 1-dehydrogenase, Glyceraldehyde-3-phosphate dehydrogenase, Peroxisomal acyl-coenzyme A oxidase 2, Bile acid-CoA: amino acid N-acyltransferase, and Isocitrate dehydrogenase 1. Connected to these proteins, we detected also proteins linked to the response to the oxidative stress such as the catalase and Glutathione S-transferase kappa 1. When analyzing the lung protein network, we mainly observed four protein clusters containing Ras-related protein, ribosomal proteins, hemoglobin, and collagen and fibrinogen proteins. Disruption of the cytoskeleton following treatment with NPs has already been reported by other in vitro studies [40,41,42,43,44,45,46,47]. However, to our knowledge, this is the first in vivo study to report cytoskeleton impairment following exposure to iron NPs in animals. In 2003, Pollak et al., reported the derangement of the cytoskeleton in the brain of patients with neurodegenerative diseases such as Down syndrome, Alzheimer’s and Pick’s diseases [48]. The effect on the cytoskeleton was marked by the variation of cytoskeletal proteins mainly actin and tubulin. For example, in the brain of rats IONPs impacted the expression of Tubulin beta-2B chain, Tropomyosin alpha-3 chain, Tubulin alpha-8 chain, Calcium/calmodulin-dependent protein kinase type IV and Tubulin beta-2A chain. The impact on the cytoskeleton could be explained by the effects that NPs can induce at the time of their internalization in the cells. Thus, depending on the size, shape, charge and surface composition, the NPs may induce significant morphological disturbances in cells and tissues. Nevertheless, even the adsorption of NPs at the cellular membranes can also induce effects without the NPs entering the cells. As in the cellular response to iron NPs by 24h treatment of SH-SY5Y cells, the most pronounced biological processes are the response to oxidative stress, inflammation, apoptosis, cytoskeletal disturbance, induction of immune system and the development of cancer [30]. Thusly, in vitro and in vivo proteomic studies are of great interest in understanding the effects and assessing toxicity in two different biological systems. Despite the different origin of the models used in the present paper and our previous proteomic paper on the in vitro effects of IONPs, our results show a few similarities. Moreover, this difference can be enriching for the evaluation of the toxicity of the NPs of iron. According to the literature, investigations of the toxicity of NPs in animal models are not sufficiently numerous as in vitro studies. Nevertheless, the in vivo studies always have a compulsory passage essentially for the test of any pharmaceutical or medical product before the clinical trials on Human.

At the cerebral level, and according to our previous results reported in 2018 [31], during the physiopathological study a disruption of the brain content of catecholamines namely dopamine and norepinephrine was detected by increasing their expression. This variation was not accompanied by a disturbance in the concentrations of trace elements including Fe, Zn, Cu and Mn in the brain. On the other hand, the proteomic study showed that physiopathological observations were more pronounced at the molecular level by the disruption of several proteins in the brain. Indeed, disturbed signaling pathways mainly involve the serotonin (5HT) signaling pathway, the “amyloid secretase” pathway of Alzheimer’s disease, the Parkinson’s disease pathway, and the dopamine and gamma-aminobutyric acid (GABA). Therefore, these results of proteomics suggest a complementary assay of other neurotransmitters namely serotonin and GABA, which would be interesting to have a global vision on the effect of iron NPs on neurotransmitter homeostasis in the brain.

Response to inflammation and oxidative stress were among the most apparent affected biological processes following the administration of iron NPs. In the liver, we observed a stimulation of the inflammation demonstrated by the increase of the level of platelets in the blood, the decrease of serum iron level and the inflammatory syndrome at the level of the liver structure. Proteomic analysis was complementary for these observations. Indeed, the proteomic study shows that the proteins known as antioxidants in response to oxidative stress have been overexpressed by the rat organism namely carbonic anhydrase 3 [49,50], Glutathione S-transferase alpha-1 and Mu2 [51,52,53], catalase [20,39,54,55,56] and isocitrate dehydrogenase [57,58,59] to fight oxidative stress and recover under normal homeostatic conditions.

The results of the proteomic study of the cerebral, hepatic and pulmonary proteomes are in agreement with our results of the physiopathological study published last year [31]. In fact, the signaling pathways found to be disturbed are related to the effects found with biochemical, hematological and histological tests, as well as the neurotransmitter and iron content in the brain following the intranasal administration of iron NPs. Although at the level of the lung, the histological examination did not highlight the inflammatory response as for the liver, the in-depth study of the pulmonary proteome suggests an inflammatory response and stimulation of the immune system by the overexpression of the proteins involved in these phenomena including annexin [60,61,62], histones H1.5 and H2A [63,64], and proteins S100-A8 and S100-A9 [65]. Deregulation of these proteins did not affect the lung tissue and/or phenotype.

## 3. Material and Methods

### 3.1. Iron Oxide Nanoparticles Synthesis and Characterization

IONPs used in this paper were gently provided by Lassaad El Mir from the Laboratory of Physics of Materials and Nanomaterials applied to the Environment at the Faculty of Sciences of Gabès, Tunisia. Suspensions of Fe_2_O_3_ NPs were prepared by a modified sol-gel method [66] under supercritical conditions of ethyl alcohol (EtOH), in which the hydrolysis proceeds slowly to release water mixture by an esterification reaction in order to control the size of the nanoparticles formed. The same nanoparticles were used in our previous paper related to the intranasal instillation exposure [31].

### 3.2. Animal Housing and Exposure Protocol

Young adult male Wistar rats (9 weeks old, ~170 g) were randomly divided into 2 groups (*n* = 6 each): one control and one treated. The animals were housed under suitable conditions of temperature (25 °C) and light (12 h:12 h light/dark cycle). All rats received water and food ad libitum. The experimental protocols were approved by the Ethic Committee of the Faculty of Sciences of Bizerte, Tunisia on February 10th, 2015 according to the Tunisian Code of Practice for the Care and Use of Animals and the Medical Ethical Committee for the Care and Use of Laboratory Animals of Pasteur Institute of Tunis (approval number: LNFP/Pro 152012) for Scientific purposes.

The treated rats received a dose of IONPs at 10 mg/kg by intranasal instillation for 7 consecutive days. Control rats were instilled with 0.9% sodium chloride solution. IONPs were suspended in purified water and then sonicated for 60 min with a hand-held sonicator (Sonics^®^). Before each administration, the solution was also vortexed for 1 min.

### 3.3. Organ/Protein Extraction and Lysis

The animals (*n* = 6 per group) were anesthetized before being sacrificed 7 days after the last injection and the different organs brain, liver and lung were extracted, from each of the 6 animals per group, to be analyzed using proteomic approach. The organs were washed with a 0.9% solution of sodium chloride and immediately soaked in liquid nitrogen before storing them at −80 °C.

From each organ of the 6 rats per group, 100 mg were ground in lysis buffer using Tissue Lyser II at a frequency of 25 Hz for a period of 25 min. Then, additional 30 min of sonication were added using the Sonics Vibracell^®^ sonicator to lyse well the tissues until a total grinding. The brain, liver and lung tissues were lysed in a lysis buffer (4% SDS, 0.1M DTT in 0.1M Tris-HCl, pH 7.6) at a ratio of 100 mg organ per 1mL lysis buffer. The organs were vortexed and incubated for 5 min at 95 °C before being lysed for 20 s with 1 sec OFF 1 sec ON at 20% amplitude using Sonics Vibracell^®^ sonicator probe. Then, the lysate was centrifuged at 14,000× *g* for 10 min at 4 °C to get rid of the cell debris.

### 3.4. Protein Quantification

At the end of the lysis step, the supernatant was recovered in a new tube and the amount of proteins from brain, liver and lung was measured using «Pierce™ BCA Protein Assay Kit» according to the instructors. Indeed, before any proteomic analysis, protein quantification is an important step that aims to standardize and fix the same amount of proteins in all samples prior to iTRAQ labeling. The protein assay by the BCA method is used for the quantification of total proteins in a sample. The principle of this method is that proteins can reduce Cu^2+^ to Cu^+^ in alkaline solution and result in purple color formation by bicinchoninic acid. Protein concentrations are determined by establishing a calibration range from BSA at 2 mg/mL. The reading is made at 562 nm using a spectrophotometer.

### 3.5. Peptide Digestion, Desalting and iTRAQ Labelling

For sample preparation, we used a modified FASP (Filter Aided Sample Preparation) method [67] adapted for iTRAQ [68]. An amount of 1 mg of protein from each of the 6 animals of each condition (control vs. exposed) was deposited in a filtration centricon Microcon YM-30 (Millipore). Washing steps were performed with 8 M urea, 0.1M Tris-HCl, pH 8.5 buffer. The cysteine residues were blocked with 12 mM MMTS (methyl methanethiosulfonate; ThermoFisher Scientific, Rockford, lL, USA) for 30 min at room temperature. Next, the proteins were digested in 0.5M TEAB, pH 8.5 by adding the trypsin/LysC Mix (Promega, Madison, WI, USA) at an enzyme/protein ratio of 1:50 overnight at 37 °C. Following the digestion, the peptides were desalted by a C18 spin column method 30 µg (89873–ThermoScientific) and then assayed by the BCA peptides method. The desalting was done according to manufacturer’s instructions. The peptide assay was done according to the Thermo Scientific Pierce™ Quantitative Colorimetric Peptide Assay kit. The reading was made at 480 nm. Peptides were then dried and labeled using iTRAQ labels for 2 h at room temperature. For each organ, two pools of peptides were extracted from the 6 biological replicates of liver, lung and brain and analyzed separately. Each organ was analyzed separately. Sample mixture of lung tissues were labeled with iTRAQ m/z 113 and 114 ion reporter tags for the control rats and iTRAQ m/z 115 and 116 ion reporter tags for the treated rats, those of liver tissues were labeled with iTRAQ m/z 117 and 118 ion reporter tags for the control rats and iTRAQ m/z 119 and 121 ion reporter tags for the treated rats. The cerebral tissues were labeled with iTRAQ m/z 114 and 115 ion reporter tags for the control rats and iTRAQ m/z 116 and 117 ion reporter tags for the treated rats. All reagents were from the iTRAQ Reagents 8 plex application kit (AB Sciex, Foster City, CA, USA).

### 3.6. Peptide Separation by OFFGEL Isoelectrofocusing Followed by Reverse Phase Nano-liquid Chromatography

Peptide fractionation according to their pI was performed on the OFFGEL 3100 Fractionator using the OFFGEL Linear pH 3-10 (Agilent Technologies) with a 24-well configuration according to the user protocol. Briefly, 90 μg of each of the iTRAQ labeled peptides were pooled and then dried in a vacuum concentrator and suspended in 3.6 mL of focused OFFGEL buffer. The IPG gel band was rehydrated and 150 μL of sample were loaded into each well. The peptides were concentrated until 50 kVh were accumulated with a maximum voltage of 8000 V, 50 μA and 200 W. After fractionation, the 24 OFFGEL fractions were desalinated using C18 ZipTips (Milipore Corp, MA, USA). The resulting fractions were collected, dried using a vacuum concentrator and maintained at −20 °C prior to nano-LC-MS/MS analysis. Peptides were separated using an Ultimate 3000 C18 reverse phase chromatography (nanoRP-LC) system controlled by Chromeleon v 7 (Dionex/LC Packings, Amsterdam, The Netherlands) and coupled to a Probot MALDI localization device controlled by the μCarrier 2.0 software (Dionex/LC Packings, Amsterdam, the Netherlands). Before the nano-LC-MS/MS, the dried fractions were resuspended in 10 µL of buffer A (98% water, 2% ACN and 0.05% TFA). Peptides were concentrated on a trapping column (C18, 3 μm, 100Å pore size, ThermoFisher Scientific) in 2% ACN and 0.05% TFA at a flow rate of 20 μL/min for 5 min prior to be separated by reverse phase chromatography (Acclaim PepMap ™ RSLC 75 μm, 15 cm, nanoViper C18, 2μm, 100Å pore size, ThermoFisher Scientific) with a binary gradient of buffer A (2% ACN and 0.05% TFA) and buffer B (80% ACN and 0.04% TFA) at a flow rate of 0.3 μL/min set up as follows: 0–5 min, 4% B; 5–35 min, 8–42% B; 35–40 min, 42–58% B; 40–50 min, 58–90% B and 50–60 min, 4% B. The entire separation run lasted 60 min. The fractions from the eluted solution were collected at a frequency of a spot per 15 s on an Opti-TOF LC/MALDI Insert 123 × 81 mm (Applied Biosystems, Foster City, CA, USA), resulting in 200 spots per fraction. The α-cyno-4-hydroxy-cinnamic acid matrix (CHCA, Sigma- Aldrich St-Louis, MO, USA) with a concentration of 2 mg/mL in 70% ACN and 0.1% TFA was added to the eluted column solution at a flow rate of 0.9 μL/min, and thus integrated in each spot of the MALDI sample plate. Each of the iTRAQ pools was injected twice in the Reverse Phase Nano-liquid Chromatography and analyzed twice by the Mass spectrometry as indicated below.

### 3.7. MALDI-TOF/TOF Analysis

MS and MS/MS analysis of peptide samples spotted by nanoLC-off-line was performed using the MALDI-TOF/TOF 4800 mass spectrometer (AB Sciex, Les Ulis, France) controlled by the Explorer Series software v 4000. The analysis was performed in positive reflector mode. MS spectra of *m*/*z* 700–3500 were acquired for each spot using the laser. The strongest ions in each MS spectrum above a Signal/Noise threshold of 10 has been selected for MS/MS analysis. The selected ions were fragmented using the Collision Induced Dissociation (CID) activation mode to obtain the corresponding MS/MS spectrum that is necessary to determine the sequence of these peptides and to quantify them.

### 3.8. Statistical Analysis of iTRAQ Data

The MS and MS/MS spectra were used for identification and relative quantification using the ProteinPilot™ v 4.0 software with the Paragon™ Algorithm (AB Sciex, Les Ulis, France). The analysis was performed with the UniProtKB/Swiss-Prot Rat database (European Institute of Bioinformatics, Hinxton, UK). In our study, the search was set to “Thorough ID” and a false discovery rate (FDR) analysis of 1% was applied. Proteins quantified with at least one peptide at the 95% peptide confidence level were included in the final set of quantified proteins. In order to perform statistical analysis for the quantified proteins we used the R package isobar (version 1.14.0). The statistical approach used through isobar was described in detail in our previous paper [29]. The analysis was performed using a normal fit and proteins which ratio had a *p*-value ratio and a *p*-value sample < 0.05 were considered significantly differentially expressed. The iTRAQ data are presented as protein ratio in the different conditions relatively to the control non-treated rats. The ratios for lung tissues Treated/Control are 115/113 and 116/114, the ratios for liver tissues are 119/117 and 121/118, and the ratios for brain tissues 116/114 and 117/115.

### 3.9. Gene Ontology, Pathway Analysis and Protein-Protein Interaction

Gene ontology analysis was performed with the list of 66, 84 and 127 dysregulated proteins respectively for liver, lung and brain tissues following IONP exposure using PANTHER (Protein ANalysisTHrough Evolutionary Relationships) Classification System Version PANTHER14.1 generated from the 2018_04 release of Reference Proteome dataset [35] http://pantherdb.org/. The version 14.1 (released 2019-03-12) contains 15,524 protein families, divided into 107,627 functionally distinct protein subfamilies [69]. The functional classification of differentially expressed proteins (DEP) regarding biological processes and protein classes are graphically illustrated in pie chart. The pathway analysis was performed using TargetMine software [34] https://targetmine.mizuguchilab.org/. Protein-protein interactions were assessed using a network analysis with STRING database Version 11.0 released in January 2019 https://string-db.org/. This version contains 24.584.628 proteins from 5.090 organisms; 3.123.056.667 interactions.

## 4. Conclusions

To conclude, the extensive toxicoproteomic study of the proteomes of the brain, liver and lung has made it possible to evaluate the toxicity of the NPs of iron at the protein and molecular levels. The results obtained provide a very important support for the estimation and understanding of the potentially toxic effects of these NPs. Indeed, to our knowledge, the effects of IONP on rat organs following intranasal exposure has never been reported in any research study before. Therefore, we believe that the present nanotoxicoproteomics study highlights new and deep effects of those nanoparticles that could not necessarily be detected by other kind of investigations. Our results are helpful for the scientific committee to better evaluate and understand the potential molecular effects of IONP on different kind of tissue (liver, lung and brain) in a model of toxicological studies such as the rat. Further in-depth studies must be conducted to minimize any risk related to exposure to these NPs iron, improve their biocompatibility and so increase their benefits.

## Figures and Tables

**Table 1:** List of signaling pathways involved in the response to IONPs at the liver level. The data were generated from PANTHER Classification system.

Pathway (Pathway Accession)	Number of Proteins	Percentage % (from 66 Proteins)	Protein Accession Number	Protein Name
Pyruvate metabolism (P02772)	2	3.1%	P12928	Pyruvate kinase PKLR
			P16638	ATP-citrate synthase
Blood coagulation (P00011)	1	1.5%	P02680	Fibrinogen gamma chain
Pentose phosphate pathway (P02762)	1	1.5%	P50137	Transketolase
Huntington disease (P00029)	3	4.6%	P04797	Glyceraldehyde-3-phosphate dehydrogenase
			P61206	ADP-ribosylation factor 3
			D3ZFQ8	Cytochrome c-1
Arginine biosynthesis (P02728)	1	1.5%	P09034	Argininosuccinate synthase
Glycolysis (P00024)	2	3.1%	P04797	Glyceraldehyde-3-phosphate dehydrogenase
			P12928	Pyruvate kinase
Parkinson disease (P00049)	1	1.5%	P63102	14-3-3 protein zeta/delta
Plasminogen activating cascade (P00050)	1	1.5%	P02680	Fibrinogen gamma chain
PI3 kinase pathway (P00048)	1	1.5%	P63102	14-3-3 protein zeta/delta
FGF signaling pathway (P00021)	1	1.5%	P63102	14-3-3 protein zeta/delta
ATP synthesis (P02721)	1	1.5%	D3ZFQ8	Cytochrome c-1
Serine glycine biosynthesis (P02776)	1	1.5%	Q5U3Z7	Serine hydroxymethyltransferase
EGF receptor signaling pathway (P00018)	1	1.5%	P63102	14-3-3 protein zeta/delta
FAS signaling pathway (P00020)	1	1.5%	D3ZFQ8	Cytochrome c-1

**Table 2:** Signaling pathways related to the nervous system identified following the dysregulation of cerebral proteins. The data were generated from PANTHER Classification system.

Pathway (Pathway Accession)	Number of Proteins	Percentage % (from 127 Proteins)	Protein Accession Number	Protein Name
5HT1 type receptor mediated signaling pathway (P04373)	1	0.8	P27791	cAMP-dependent protein kinase catalytic subunit alpha
Alzheimer disease-amyloid secretase pathway (P00003)	2	1.6	P08592	Amyloid-beta A4 protein
			P49186	Mitogen-activated protein kinase 9
Beta1 adrenergic receptor signaling pathway (P04377)	1	0.8	P27791	cAMP-dependent protein kinase catalytic subunit alpha
Beta2 adrenergic receptor signaling pathway (P04378)	1	0.8	P27791	cAMP-dependent protein kinase catalytic subunit alpha
Dopamine receptor mediated signaling pathway (P05912)	3	2.4	Q6J4I0	Protein phosphatase 1 regulatory subunit 1B
			P27791	cAMP-dependent protein kinase catalytic subunit alpha
			P19627	Guanine nucleotide-binding protein G(z) subunit alpha
Muscarinic acetylcholine receptor 2 and 4 signaling pathway	1	0.8	P27791	cAMP-dependent protein kinase catalytic subunit alpha
Enkephalin release (P05913)	1	0.8	P27791	cAMP-dependent protein kinase catalytic subunit alpha
GABA-B receptor II signaling (P05731)	1	0.8	P27791	cAMP-dependent protein kinase catalytic subunit alpha
Gamma-aminobutyric acid synthesis (P04384)	1	0.8	P51650	Succinate-semialdehyde dehydrogenase, mitochondrial
Gonadotropin-releasing hormone receptor pathway (P6664)	3	2.4	P49186	Mitogen-activated protein kinase 9
			P14668	Annexin A5
			P63055	Calmodulin regulator protein PCP4
Heterotrimeric G-protein signaling pathway-Gi alpha and Gs alpha mediated pathway (P00026)	1	0.8	P27791	cAMP-dependent protein kinase catalytic subunit alpha
Heterotrimeric G-protein signaling pathway-rod outer segment phototransduction (P00028)	1	0.8	P27791	cAMP-dependent protein kinase catalytic subunit alpha
Metabotropic glutamate receptor group I pathway (P00041)	1	0.8	P27791	cAMP-dependent protein kinase catalytic subunit alpha
Metabotropic glutamate receptor group II pathway (P00040)	1	0.8	P27791	cAMP-dependent protein kinase catalytic subunit alpha
Metabotropic glutamate receptor group III pathway (P00039)	1	0.8	P27791	cAMP-dependent protein kinase catalytic subunit alpha
Huntington disease (P00029)	4	3.2	P49186	Mitogen-activated protein kinase 9
			Q3KRE8	Tubulin beta-2B chain
			P85108	Tubulin beta-2A chain
			Q07009	Calpain-2 catalytic subunit
Nicotine pharmacodynamics pathway (P06587)	2	1.6	Q6J4I0	Protein phosphatase 1 regulatory subunit 1B
			P27791	cAMP-dependent protein kinase catalytic subunit alpha
Nicotinic acetylcholine receptor signaling pathway (P00044)	1	0.8	Q62812	Myosin-9
Parkinson disease (P00049)	2	1.6	P49186	Mitogen-activated protein kinase 9
			P37377	Alpha-synuclein

**Table 3:** Signaling pathways related to inflammatory, immune, apoptotic and cancerous processes identified by dysregulation of cerebral Proteins. The data were generated from PANTHER Classification system.

Pathway (Pathway Accession)	Number of Proteins	Percentage % (from 127 Proteins)	Protein Accession Number	Protein Name
Apoptosis signaling pathway (P00006)	1	0.8	P49186	Mitogen-activated protein kinase 9
Toll receptor signaling pathway (P00054)	1	0.8	P49186	Mitogen-activated protein kinase 9
B cell activation (P00010)	1	0.8	P49186	Mitogen-activated protein kinase 9
T cell activation (P00053)	1	0.8	P49186	Mitogen-activated protein kinase 9
Oxidative stress response (P00046)	1	0.8	P49186	Mitogen-activated protein kinase 9
Inflammation mediated by chemokine and cytokine signaling pathway (P00031)	2	1.6	P49186	Mitogen-activated protein kinase 9
			P27791	cAMP-dependent protein kinase catalytic subunit alpha
Integrin signaling pathway (P00034)	1	0.8	P49186	Mitogen-activated protein kinase 9
Interferon-gamma signaling pathway (P00035)	1	0.8	P49186	Mitogen-activated protein kinase 9
Histamine H2 receptor mediated signaling pathway (P04386)	1	0.8	P27791	cAMP-dependent protein kinase catalytic subunit alpha
Ras Pathway (P04393)	1	0.8	P49186	Mitogen-activated protein kinase 9
CCKR signaling map (P06959)	3	2.4	P49186	Mitogen-activated protein kinase 9
			P13234	Calcium/calmodulin-dependent protein kinase type IV
			P27791	cAMP-dependent protein kinase catalytic subunit alpha
Cytoskeletal regulation by Rho GTPase (P00016)	3	2.4	Q3KRE8	Tubulin beta-2B chain
			Q62812	Myosin-9
			P85108	Tubulin beta-2A chain
De novo purine biosynthesis (P02738)	2	1.6	O35567	Bifunctional purine biosynthesis protein PURH
			P19804	Nucleoside diphosphate kinase B
De novo pyrimidine deoxyribonucleotide biosynthesis (P02739)	1	0.8	P19804	Nucleoside diphosphate kinase B
De novo pyrimidine ribonucleotides biosythesis (P02740)	1	0.8	P19804	Nucleoside diphosphate kinase B

**Table 4:** Examples of dysregulated proteins in the brain following exposure to Iron NPs and related biological processes. The data were generated from TargetMine software: Biological process name, protein name, protein accession number, ratio exposed (Exp)/control (Ctr), *p*-value ratio. Up-regulated proteins are highlighted in green color and down-regulated proteins in red color.

Biological Process	Accession Number	Ratio Exp/Ctr	*p*-Value
**Response to oxidative stress**			
Glutathione S-transferase Yb-3	P08009	1.62	<0.001
NADH dehydrogenase	B0BNE6	2.30	0.027
**Inflammation process**			
Annexin A5	P14668	1.37	0.02
**Cancer: angio/lymphangiogenesis**			
Ezrin	P31977	1.31	0.0038
Malignant T-cell-amplified sequence 1	Q4G009	1.30	0.012
**Neuronal damage related to Alzheimer Disease**			
Neuronal pentraxin-1	P47971	1.36	0.009
**Adherence, neuronal differentiation and signal transduction**			
Disks large homolog 1	Q62696	0.56	0.025
Cell cycle exit and neuronal differentiation protein1	Q5FVI4	0.73	0.015

**Table 5:** Examples of dysregulated proteins in the liver following exposure to iron NPs and the corresponding biological processes. The data were generated from TargetMine software: Biological process name, protein name, protein accession number, ratio exposed (Exp)/control (Ctr), *p*-value ratio. Up-regulated proteins are highlighted in green color and down-regulated proteins in red color.

Biological Process	Accession Number	Ratio Exp/Ctr	*p*-Value
**Response to oxidative stress**			
Carbonic anhydrase 3	P14141	2.07	<0.001
Glutathione S-transferase alpha-1	P00502	1.30	<0.001
Glutathione S-transferase Mu 2	P08010	1.30	0.005
Catalase	P04762	1.26	0.015
Isocitrate dehydrogenase	P41562	1.20	0.012
**Immune system**			
Ig gamma-1 chain C region	P20759	1.56	0.029
**Cancer metabolism**			
Sulfotransferase 1C1	P50237	1.66	<0.001
**Hepatic lipid metabolism**			
Vigilin	Q9Z1A6	1.47	0.012
**Fatty Acide Metabolism**			
Long-chain-fatty-acid--CoA ligase 5	O88813	0.73	0.022
Fatty acid synthase	P12785	0.63	<0.001
**Glycolysis/Gluconeogenesis**			
ATP-citrate synthase	P16638	0.59	<0.001
Glyceraldehyde-3-phosphate dehydrogenase	P04797	0.79	0.025
Glucose-6-phosphate 1-dehydrogenase	P05370	0.60	0.008
Pyruvate kinase	P12928	0.66	<0.001
Cytochrome C1	D3ZFQ8	0.82	0.043
**Oxidative Phosphorylation**			
Cytochrome c oxidase COX 7B	P80431	0.65	0.046

**Table 6:** Examples of dysregulated proteins in the lung following exposure to Iron NPs and corresponding biological processes. The data were generated from TargetMine software: Biological process name, protein name, protein accession number, ratio exposed (Exp)/control (Ctr), *p*-value ratio. Up-regulated proteins are highlighted in green color and down-regulated proteins in red color.

Biological Process	Accession Number	Ratio Exp/ctr	*p*-Value
**Response to oxidative stress**			
Superoxide dismutase	P07895	1.48	0.005
Peroxiredoxin-6	O35244	1.26	0.049
Redox-regulatory protein FAM213A	Q6AXX6	1.22	0.013
**Immune system stimulation and response to inflammation**			
Annexin	Q5XI77	1.54	0.021
Histone H1.5	D3ZBN0	1.35	0.025
Histone H2A	Q6I8Q6	1.75	0.021
Protein S100-A8	P50115	1.30	0.009
Protein S100-A9	P50116	1.65	<0.001
Receptor-type tyrosine-protein phosphatase C	P04157	1.71	0.004
Redox-regulatory protein FAM213A	Q6AXX6	1.22	0.013
**Cell death**			
Programmed cell death 6	G3V7W1	1.23	0.045
**Iron metabolism**			
Ferritin heavy chain	P19132	1.36	<0.001
**Cell adhesion and cancer progress**			
Podocalyxin	Q9WTQ2	1.96	0.002
**Metabolism**			
Succinate dehydrogenase	P21913	0.71	0.005
Phosphoglycerate kinase 1	P16617	0.79	0.044
**Oxygen transport (lung to tissues)**			
Hemoglobin subunit alpha-1/2	P01946	0.37	<0.001
Hemoglobin subunit beta-1 and beta-2	P02091	0.77	<0.001
**Hemostasis**			
Fibrinogen gamma chain	P02680	0.79	0.008
Fibrinogen beta chain	P14480	0.81	0.017
**Cell proliferation and differentiation**			
Thymosin beta-4	P62329	0.61	<0.001

**Table 7:** List of the dysregulated cytoskeleton proteins in the brain, liver and lung following exposure to Iron NPs.

Organ	Accession Number	Protein Name	Panther Protein Class
Brain	Q3KRE8	Tubulin beta-2B chain	Tubulin
	P31977	Ezrin	Actin family cytoskeletal protein
	Q63610	Tropomyosin alpha-3 chain	Actin binding motor protein
	Q6AY56	Tubulin alpha-8 chain	Tubulin
	P13234	Calcium/calmodulin-dependent protein kinase type IV	Non-motor microtubule binding protein Non-receptor serine/threonine protein kinase
	P85108	Tubulin beta-2A chain	Tubulin
Liver	Q7M0E3	Destrin	Non-motor actin binding protein
	P63029	Translationally-controlled tumor protein	Ton-motor actin binding protein
	P09495	Tropomyosin alpha-4 chain	Actin binding motor protein
Lung	P47875	Cysteine and glycine-rich protein 1	Actin family cytoskeletal protein
	Q63598	Plastin-3	Non-motor actin binding protein
	Q63355	Unconventional myosin-Ic	G-protein modulatoractin binding motor proteincell junction protein

## Supplementary Materials

Supplementary materials can be found at https://www.mdpi.com/1422-0067/20/20/5186/s1.
