# Supplementary material for: Effects of Iron Oxide Nanoparticles (γ-Fe2O3) on Liver, Lung and Brain Proteomes following Sub-Acute Intranasal Exposure: A New Toxicological Assessment in Rat Model Using iTRAQ-Based Quantitative Proteomics
Source: Int J Mol Sci. 2019 Oct 19;20(20):5186. doi: 10.3390/ijms20205186 (PMC6829235; doi:10.3390/ijms20205186)
Supplement: Supplementary file 1 [file ijms-20-05186-s001.pdf]

Supplementary material:

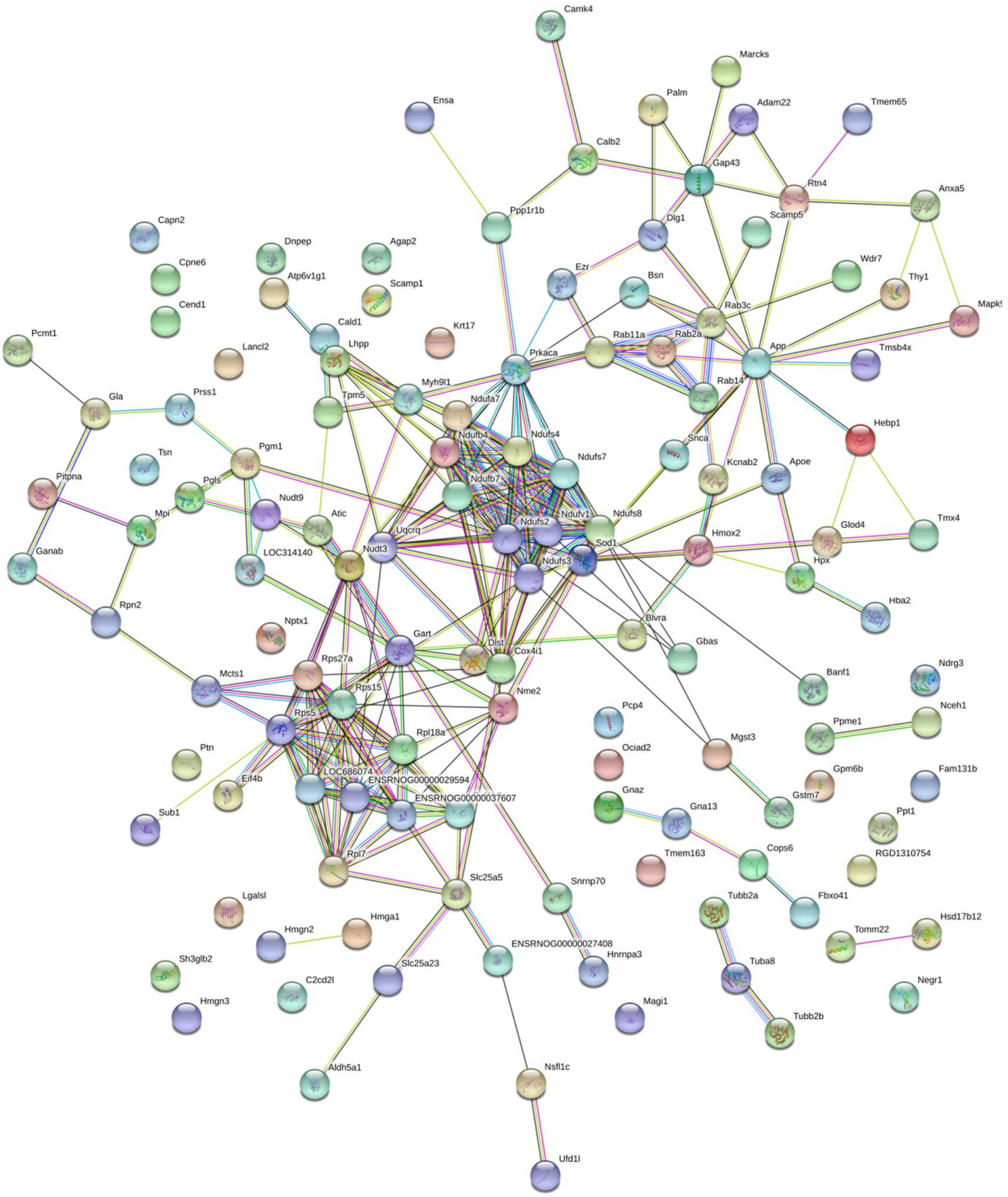

Figure S1. Protein-Protein Interactions of the 127 Dysregulated proteins in brain tissues following IONP exposure. The graph was generated from STRING database. 125 nodes, 183 edges, 2.93 Average node degree, 0.411 Avg. local clustering coefficient, 122 Expected number of edges, PPI enrichment p-value 1.83e-07.

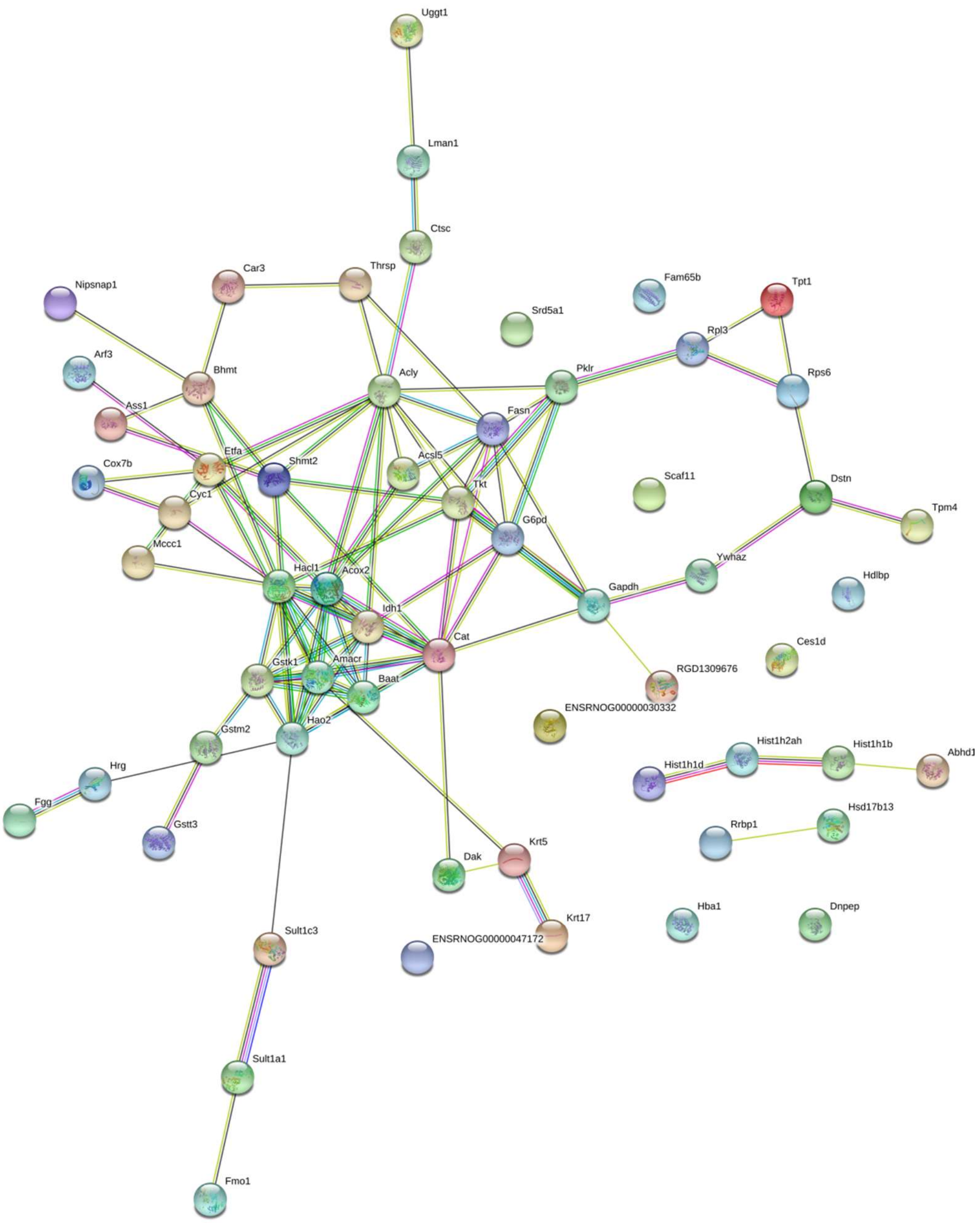

Figure S2. Protein-Protein Interactions of the 66 Dysregulated proteins in liver tissues following IONP exposure. The graph was generated from STRING database. 61 nodes, 103 edges, 3.38 Average node degree, 0.412 Avg. local clustering coefficient, 22 Expected number of edges, PPI enrichment p-value < 1.0e-16.

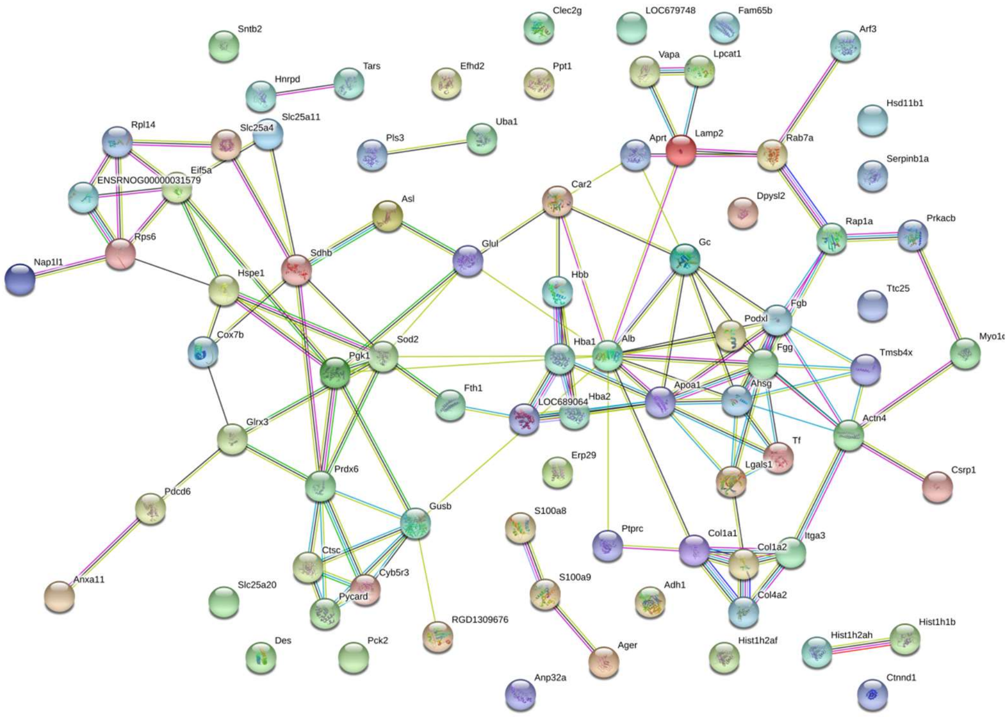

Figure S3. Protein-Protein Interactions of the 84 Dysregulated proteins in lung tissues following IONP exposure. The graph was generated from STRING database. 82 nodes, 125 edges, 3.05 Average node degree, 0.422 Avg. local clustering coefficient, 64 Expected number of edges, PPI enrichment p-value 1.07e-11.

Table S1. List of all dysregulated proteins in brain tissues following IONP exposure. A) Down regulated proteins and B) Upregulated proteins. Statistically significant iTRAQ ratios (p-value ratio and p-value sample≤0.05) for the 127 proteins that are dysregulated.

A)

Accession	Name	Description	Gene	Peptide Count	Spectral Count	Sequence Coverage %	Ratio	*P*-Value Ratio	*P*-Value Sample	Log 10 Ratio
P00762	TRY1_RAT	Anionic trypsin-1	Prss1	1	8	8.13	0.526	0.001	0.004	-0.279
P08592	A4_RAT	Amyloid beta A4 protein	App	1	2	1.17	0.546	0.010	0.004	-0.263
Q62696	DLG1_RAT	Disks large homolog 1	Dlg1	1	1	1.32	0.558	0.026	0.005	-0.253
B2RZ74	B2RZ74_RAT	Protein Snrnp70	Snrnp70	1	1	2.88	0.566	0.024	0.005	-0.247
Q3KRE8	TBB2B_RAT	Tubulin beta-2B chain	Tubb2b	1	10	2.7	0.568	0.000	0.005	-0.246
Q66H40	HMGN3_RAT	High mobility group nucleosome-binding domain-containing protein 3	Hmgn3	1	4	15.79	0.583	0.001	0.006	-0.234
B4F7C7	B4F7C7_RAT	Hebp1 protein	Hebp1	2	4	6.32	0.592	0.031	0.009	-0.228
Q62736	CALD1_RAT	Non-muscle caldesmon	Cald1	2	4	3.77	0.601	0.000	0.008	-0.222
P18437	HMGN2_RAT	Non-histone chromosomal protein HMG-17	Hmgn2	1	8	16.67	0.603	0.004	0.008	-0.219
Q9ES53	UFD1_RAT	Ubiquitin fusion degradation protein 1 homolog	Ufd1l	1	2	5.21	0.615	0.003	0.008	-0.211
F1LXC7	F1LXC7_RAT	Protein LOC100362814	LOC100362814	1	1	0.82	0.640	0.021	0.011	-0.194
Q4L1J4	MAGI1_RAT	Membrane-associated guanylate kinase, WW and PDZ domain-containing protein 1	Magi1	1	1	1.12	0.641	0.022	0.011	-0.193
Q8K585	HMGA1_RAT	High mobility group protein HMG-I/HMG-Y	Hmga1	1	2	23.36	0.654	0.026	0.011	-0.184
F1LPG5	F1LPG5_RAT	Protein Ndufb4	Ndufb4	3	10	8.53	0.673	0.008	0.014	-0.172
P62824	RAB3C_RAT	Ras-related protein Rab-3C	Rab3c	1	4	6.17	0.677	0.003	0.017	-0.170
P25235	RPN2_RAT	Dolichyl-diphosphooligosaccharide--protein glycosyltransferase subunit 2	Rpn2	1	3	2.69	0.679	0.003	0.015	-0.168
P02650	APOE_RAT	Apolipoprotein E	Apoe	3	8	1.92	0.684	0.000	0.015	-0.165
A9UMV9	A9UMV9_RAT	Ndufa7 protein	Ndufa7	3	10	9.82	0.689	0.002	0.014	-0.162

Accession	Name	Description	Gene	Peptide Count	Spectral Count	Sequence coverage %	Ratio	p-value Ratio	p-value Sample	Log 10 Ratio
Q8CGU4	AGAP2_RAT	Arf-GAP with GTPase, ANK repeat and PH domain-containing protein 2	Agap2	3	4	1.43	0.689	0.012	0.016	-0.162
P62329	TYB4_RAT	Thymosin beta-4	Tmsb4x	5	11	29.55	0.691	0.001	0.020	-0.161
P30009	MARCS_RAT	Myristoylated alanine-rich C-kinase substrate	Marcks	7	47	4.85	0.694	0.007	0.020	-0.158
Q9Z0J8	NEGR1_RAT	Neuronal growth regulator 1	Negr1	1	9	3.74	0.699	0.003	0.020	-0.155
P38652	PGM1_RAT	Phosphoglucomutase-1	Pgm1	5	19	2.31	0.700	0.011	0.031	-0.155
F1LQZ0	F1LQZ0_RAT	Protein Tmem65	Tmem65	1	3	5.41	0.701	0.007	0.021	-0.154
D3ZT20	D3ZT20_RAT	Protein Fbxo41	Fbxo41	1	2	1.84	0.705	0.044	0.021	-0.152
P85108	TBB2A_RAT	Tubulin beta-2A chain	Tubb2a	2	45	2.7	0.707	0.000	0.021	-0.151
B2GUV5	B2GUV5_RAT	ATPase, H transporting, lysosomal V1 subunit G1	Atp6v1g1	1	4	9.32	0.707	0.004	0.019	-0.151
P62845	RS15_RAT	40S ribosomal protein S15	Rps15	1	8	8.97	0.711	0.019	0.025	-0.148
P85971	6PGL_RAT	6-phosphogluconolactonase	Pgls	2	4	6.23	0.719	0.000	0.022	-0.143
P56603	SCAM1_RAT	Secretory carrier-associated membrane protein 1	Scamp1	3	7	3.85	0.724	0.008	0.021	-0.141
P62494	RB11A_RAT	Ras-related protein Rab-11A	Rab11a	3	5	3.7	0.726	0.004	0.025	-0.139
Q63396	TCP4_RAT	Activated RNA polymerase II transcriptional coactivator p15	Sub1	2	5	7.87	0.727	0.012	0.027	-0.139
Q9JKE3	SCAM5_RAT	Secretory carrier-associated membrane protein 5	Scamp5	1	6	4.68	0.731	0.006	0.026	-0.136
P47728	CALB2_RAT	Calretinin	Calb2	1	4	4.43	0.732	0.017	0.022	-0.135
Q5FVI4	CEND_RAT	Cell cycle exit and neuronal differentiation protein 1	Cend1	9	43	5.37	0.734	0.015	0.037	-0.134
P13234	KCC4_RAT	Calcium/calmodulin-dependent protein kinase type IV	Camk4	3	6	2.53	0.737	0.002	0.028	-0.132
Q5RJN0	Q5RJN0_RAT	NADH dehydrogenase	Ndufs7	4	15	6.88	0.743	0.011	0.044	-0.129
P07936	NEUM_RAT	Neuromodulin	Gap43	9	29	11.95	0.743	0.020	0.028	-0.129

Accession	Name	Description	Gene	Peptide Count	Spectral Count	Sequence coverage %	Ratio	p-value Ratio	p-value Sample	Log 10 Ratio
B5DEN5	B5DEN5_RAT	Eukaryotic translation elongation factor 1 beta 2	Eef1b2	2	4	4	0.745	0.000	0.030	-0.128
A9CMA6	TM163_RAT	Transmembrane protein 163	Tmem163	1	3	3.47	0.749	0.034	0.048	-0.126
Q9JK11	RTN4_RAT	Reticulon-4	Rtn4	5	11	1.12	0.749	0.013	0.031	-0.125
P60841	ENSA_RAT	Alpha-endosulfine	Ensa	4	10	14.88	0.750	0.009	0.034	-0.125
P62982	RS27A_RAT	Ubiquitin-40S ribosomal protein S27a	Rps27a	6	69	9.62	0.750	0.014	0.029	-0.125
Q568Z1	F131B_RAT	Protein FAM131B	Fam131b	2	4	4.22	0.754	0.013	0.043	-0.123
Q5XIF3	NDUS4_RAT	NADH dehydrogenase [ubiquinone] iron-sulfur protein 4, mitochondrial	Ndufs4	2	6	5.71	0.755	0.001	0.035	-0.122
Q920Q0	PALM_RAT	Paralemmin-1	Palm	1	6	4.96	0.756	0.012	0.030	-0.122
Q6P7R8	DHB12_RAT	Very-long-chain 3-oxoacyl-CoA reductase	Hsd17b12	2	7	4.81	0.757	0.034	0.022	-0.121
O35987	NSF1C_RAT	NSFL1 cofactor p47	Nsfl1c	2	3	4.32	0.760	0.018	0.033	-0.119
M0R665	M0R665_RAT	Uncharacterized protein		2	4	7.05	0.761	0.012	0.040	-0.119

Accession	Name	Description	Gene	Peptide Count	Spectral Count	Sequence coverage %	Ratio	p-value Ratio	p-value Sample	Log 10 Ratio
Q63610	TPM3_RAT	Tropomyosin alpha-3 chain	Tpm3	3	11	2.82	0.762	0.003	0.041	-0.118
E9PT79	E9PT79_RAT	Protein Tsn	Tsn	1	4	3.6	0.763	0.005	0.035	-0.117
Q75Q41	TOM22_RAT	Mitochondrial import receptor subunit TOM22 homolog	Tomm22	3	6	8.45	0.763	0.037	0.031	-0.117
P07632	SODC_RAT	Superoxide dismutase [Cu-Zn]	Sod1	5	21	7.14	0.764	0.012	0.035	-0.117
P63090	PTN_RAT	Pleiotrophin	Ptn	1	4	6.55	0.766	0.003	0.036	-0.116
B2GV54	NCEH1_RAT	Neutral cholesterol ester hydrolase 1	Nceh1	3	11	3.92	0.767	0.034	0.047	-0.115
P63055	PCP4_RAT	Purkinje cell protein 4	Pcp4	3	14	16.13	0.769	0.006	0.040	-0.114
Q6DGG0	PPID_RAT	Peptidyl-prolyl cis-trans isomerase D	Ppid	3	8	2.16	0.771	0.003	0.036	-0.113
Q6URK4	ROA3_RAT	Heterogeneous nuclear ribonucleoprotein A3	Hnrnpa3	9	20	3.43	0.776	0.046	0.050	-0.110
O88778	BSN_RAT	Protein bassoon	Bsn	21	66	0.66	0.778	0.023	0.041	-0.109
P10888	COX41_RAT	Cytochrome c oxidase subunit 4 isoform 1, mitochondrial	Cox4i1	9	45	4.73	0.783	0.042	0.047	-0.106
Q5RKG9	Q5RKG9_RAT	Eukaryotic translation initiation factor 4B	Eif4b	2	5	2.62	0.784	0.001	0.045	-0.106
Q6J4I0	PPR1B_RAT	Protein phosphatase 1 regulatory subunit 1B	Ppp1r1b	3	20	9.76	0.787	0.017	0.050	-0.104
P05426	RL7_RAT	60S ribosomal protein L7	Rpl7	6	11	4.23	0.789	0.013	0.042	-0.103
P16446	PIPNA_RAT	Phosphatidylinositol transfer protein alpha isoform	Pitpna	6	14	3.32	0.790	0.022	0.049	-0.102

B)

Accession	Name	Description	Gene	Peptide Count	Spectral Count	Sequence coverage %	Ratio	p-value Ratio	p-value Sample	Log 10 Ratio
D3ZAN3	D3ZAN3_RAT	Alpha glucosidase 2 alpha neutral subunit	Ganab	6	10	1.38	1.207	0.026	0.047	0.082
Q5I0D1	GLOD4_RAT	Glyoxalase domain-containing protein 4	Glod4	2	4	2.68	1.227	0.027	0.037	0.089
P05712	RAB2A_RAT	Ras-related protein Rab-2A	Rab2a	4	14	6.13	1.237	0.050	0.038	0.092
Q7TQ16	QCR8_RAT	Cytochrome b-c1 complex subunit 8	Uqcrq	4	13	9.76	1.262	0.024	0.040	0.101
Q5PPJ9	SHLB2_RAT	Endophilin-B2	Sh3glb2	6	21	3.71	1.271	0.030	0.036	0.104
P22062	PIMT_RAT	Protein-L-isoaspartate(D-aspartate) O-methyltransferase	Pcmt1	4	7	7.49	1.292	0.047	0.021	0.111
Q4G009	MCTS1_RAT	Malignant T-cell-amplified sequence 1	Mcts1	1	5	10.44	1.305	0.013	0.039	0.115
P49186	MK09_RAT	Mitogen-activated protein kinase 9	Mapk9	1	7	3.07	1.306	0.001	0.041	0.116
P51650	SSDH_RAT	Succinate-semialdehyde dehydrogenase, mitochondrial	Aldh5a1	7	16	2.1	1.310	0.013	0.037	0.117
P31977	EZRI_RAT	Ezrin	Ezr	2	3	1.19	1.312	0.004	0.041	0.118
D3ZN79	D3ZN79_RAT	Protein LOC686074	LOC686074	1	4	8.13	1.315	0.009	0.038	0.119
P19804	NDKB_RAT	Nucleoside diphosphate kinase B	Nme2	5	15	9.21	1.323	0.000	0.038	0.122
Q01205	ODO2_RAT	Dihydrolipoyllysine-residue succinyltransferase component of 2-oxoglutarate dehydrogenase complex, mitochondrial	Dlst	6	21	1.76	1.331	0.005	0.027	0.124
Q5XIG0	NUDT9_RAT	ADP-ribose pyrophosphatase, mitochondrial	Nudt9	1	3	2.57	1.339	0.005	0.029	0.127
Q09073	ADT2_RAT	ADP/ATP translocase 2	Slc25a5	9	46	3.69	1.341	0.014	0.028	0.127
D3ZI16	D3ZI16_RAT	COP9	Cops6	2	5	3.49	1.348	0.034	0.038	0.130
O35567	PUR9_RAT	Bifunctional purine biosynthesis protein PURH	Atic	1	4	1.86	1.356	0.035	0.036	0.132
P47971	NPTX1_RAT	Neuronal pentraxin-1	Nptx1	1	6	1.85	1.364	0.009	0.030	0.135

Accession	Name	Description	Gene	Peptide Count	Spectral Count	Sequence coverage %	Ratio	p-value Ratio	p-value Sample	Log 10 Ratio
Q9ERH3	WDR7_RAT	WD repeat-containing protein 7	Wdr7	3	7	0.87	1.366	0.001	0.023	0.135
Q6Q7Y5	GNA13_RAT	Guanine nucleotide-binding protein subunit alpha-13	Gna13	2	5	2.65	1.372	0.002	0.025	0.137
P14668	ANXA5_RAT	Annexin A5	Anxa5	3	15	2.51	1.378	0.021	0.029	0.139
D3ZLT1	D3ZLT1_RAT	NADH dehydrogenase	Ndufb7	3	15	6.57	1.387	0.000	0.019	0.142
Q5RK08	Q5RK08_RAT	Glioblastoma amplified sequence	Gbas	3	6	5.69	1.393	0.002	0.021	0.144
D4ADS4	D4ADS4_RAT	Protein Mgst3	Mgst3	2	9	8.55	1.396	0.018	0.028	0.145
Q9JJK1	GPM6B_RAT	Neuronal membrane glycoprotein M6-b	Gpm6b	1	9	4.91	1.397	0.000	0.018	0.145
P62483	KCAB2_RAT	Voltage-gated potassium channel subunit beta-2	Kcnab2	4	10	1.91	1.404	0.005	0.013	0.147
Q68FX1	MPI_RAT	Mannose-6-phosphate isomerase	Mpi	1	3	3.55	1.412	0.016	0.019	0.150
P01830	THY1_RAT	Thy-1 membrane glycoprotein	Thy1	6	45	6.21	1.421	0.012	0.018	0.153
Q566C7	NUDT3_RAT	Diphosphoinositol polyphosphate phosphohydrolase 1	Nudt3	1	7	10.12	1.437	0.026	0.024	0.157
Q5U2P5	Q5U2P5_RAT	Protein C2cd2l	C2cd2l	1	3	1.42	1.453	0.001	0.012	0.162
P45479	PPT1_RAT	Palmitoyl-protein thioesterase 1	Ppt1	2	5	3.92	1.455	0.009	0.015	0.163
P23711	HMOX2_RAT	Heme oxygenase 2	Hmox2	2	4	5.08	1.459	0.010	0.016	0.164
P02401	RLA2_RAT	60S acidic ribosomal protein P2	Rplp2	1	4	16.52	1.462	0.005	0.014	0.165
P19627	GNAZ_RAT	Guanine nucleotide-binding protein G(z) subunit alpha	Gnaz	3	13	3.94	1.464	0.002	0.014	0.166
M0R5P8	M0R5P8_RAT	Protein Adam22	Adam22	4	10	1.55	1.467	0.016	0.010	0.166
Q07009	CAN2_RAT	Calpain-2 catalytic subunit	Capn2	2	4	1.57	1.475	0.027	0.014	0.169
Q6AYR2	NDRG3_RAT	Protein NDRG3	Ndrg3	2	8	4.53	1.477	0.004	0.015	0.169
B4F7A3	B4F7A3_RAT	Galectin	Lgalsl	2	8	5.81	1.480	0.037	0.027	0.170
P20059	HEMO_RAT	Hemopexin	Hpx	3	6	1.74	1.481	0.000	0.017	0.171
P46844	BIEA_RAT	Biliverdin reductase A	Blvra	1	3	4.41	1.487	0.001	0.015	0.172
Q4V8H5	Q4V8H5_RAT	Aspartyl aminopeptidase	Dnpep	2	4	2.32	1.510	0.008	0.013	0.179
D4A4Q4	D4A4Q4_RAT	Protein Ociad2	Ociad2	1	8	9.85	1.515	0.017	0.008	0.181

Accession	Name	Description	Gene	Peptide Count	Spectral Count	Sequence coverage %	Ratio	p-value Ratio	p-value Sample	Log 10 Ratio
P27791	KAPCA_RAT	cAMP-dependent protein kinase catalytic subunit alpha	Prkaca	1	6	3.42	1.521	0.000	0.010	0.182
Q9R1T1	BAF_RAT	Barrier-to-autointegration factor	Banf1	1	7	13.48	1.536	0.001	0.011	0.187
Q4FZT2	PPME1_RAT	Protein phosphatase methylesterase 1	Ppme1	1	9	3.37	1.553	0.006	0.010	0.191
Q68FQ9	Q68FQ9_RAT	LanC lantibiotic synthetase component C-like 2	Lancl2	3	4	2	1.577	0.001	0.009	0.198
G3V912	G3V912_RAT	Protein Tmx4	Tmx4	1	2	3.87	1.583	0.022	0.010	0.200
Q5I0D5	LHPP_RAT	Phospholysine phosphohistidine inorganic pyrophosphate phosphatase	Lhpp	1	3	4.44	1.610	0.027	0.008	0.207
P08009	GSTM4_RAT	Glutathione S-transferase Yb-3	Gstm3	6	15	4.13	1.619	0.000	0.008	0.209
P61107	RAB14_RAT	Ras-related protein Rab-14	Rab14	3	6	7.44	1.692	0.000	0.006	0.228
P62718	RL18A_RAT	60S ribosomal protein L18a	Rpl18a	1	3	5.11	1.718	0.000	0.006	0.235
Q6AY56	TBA8_RAT	Tubulin alpha-8 chain	Tuba8	1	3	3.56	1.788	0.000	0.004	0.252
D3ZJF9	D3ZJF9_RAT	Protein Gla	Gla	2	3	2.62	1.800	0.007	0.004	0.255
Q6IFU8	K1C17_RAT	Keratin, type I cytoskeletal 17	Krt17	2	7	1.62	1.810	0.000	0.003	0.258
D4ACG7	D4ACG7_RAT	Protein Cpne6	Cpne6	1	3	3.41	1.858	0.000	0.003	0.269
Q62812	MYH9_RAT	Myosin-9	Myh9	2	2	0.71	1.978	0.000	0.003	0.296
M0R4V4	M0R4V4_RAT	Protein Slc25a23	Slc25a23	1	1	2.78	2.280	0.023	0.001	0.358
B0BNE6	B0BNE6_RAT	NADH dehydrogenase	Ndufs8	1	2	4.25	2.307	0.027	0.001	0.363

Table S2. List of all dysregulated proteins in liver tissues following IONP exposure. A) Down regulated proteins and B) Upregulated proteins.Statistically significant iTRAQ ratios (p-value ratio and p-value sample≤0.05) for the 66 proteins that are dysregulated.

A)

Accession	Name	Description	Gene	Peptide Count	Spectral Count	Sequence coverage %	Ratio	p-value Ratio	p-value Sample	Log 10 Ratio
Q6IFU8	K1C17_RAT	Keratin, type I cytoskeletal 17	Krt17	1	1	2.54	0.312	0.005	0.000	-0.506
Q99PS8	HRG_RAT	Histidine-rich glycoprotein	Hrg	2	3	2.67	0.575	0.003	0.001	-0.241
Q6P6Q2	K2C5_RAT	Keratin, type II cytoskeletal 5	Krt5	1	1	1.39	0.578	0.015	0.002	-0.238

D3ZBN0	H15_RAT	Histone H1.5	Hist1h1b	3	5	4.95	0.589	0.018	0.003	-0.230
P0C170	H2A1E_RAT	Histone H2A type 1-E		1	6	5.38	0.592	0.000	0.002	-0.228
P16638	ACLY_RAT	ATP-citrate synthase	Acly	18	48	1.73	0.597	0.001	0.002	-0.224
P05370	G6PD_RAT	Glucose-6-phosphate 1-dehydrogenase	G6pdx	2	3	1.94	0.605	0.008	0.003	-0.218
P24008	S5A1_RAT	3-oxo-5-alpha-steroid 4-dehydrogenase 1	Srd5a1	1	2	3.47	0.628	0.044	0.003	-0.202
P12785	FAS_RAT	Fatty acid synthase	Fasn	34	131	0.32	0.631	0.000	0.003	-0.200
A0A0G2K1A2	A0A0G2K1A2_RAT	Myeloperoxidase	Mpo	2	4	1.00	0.640	0.043	0.005	-0.194
P80431	COX7B_RAT	Cytochrome c oxidase subunit 7B, mitochondrial	Cox7b	1	2	10.00	0.650	0.046	0.005	-0.187
A0A0G2K916	A0A0G2K916_RAT	Ankyrin repeat and KH domain-containing 1		1	2	1.00	0.658	0.045	0.004	-0.182
P12928	KPYR_RAT	Pyruvate kinase PKLR	Pklr	8	20	1.92	0.665	0.000	0.004	-0.177
Q5M875	DHB13_RAT	17-beta-hydroxysteroid dehydrogenase 13	Hsd17b13	3	4	4.33	0.668	0.008	0.004	-0.175
O09171	BHMT1_RAT	Betaine--homocysteine S-methyltransferase 1	Bhmt	17	134	5.41	0.681	0.000	0.005	-0.167
F1M853	F1M853_RAT	Protein Rrbp1	Rrbp1	6	9	0.96	0.683	0.001	0.005	-0.165
P15865	H14_RAT	Histone H1.4	Hist1h1e	2	8	4.11	0.701	0.048	0.007	-0.154
P13803	ETFA_RAT	Electron transfer flavoprotein subunit alpha, mitochondrial	Etfa	5	15	4.80	0.724	0.002	0.010	-0.140

**Accession**	**Name**	**Description**	**Gene**	**Peptide Count**	**Spectral Count**	**Sequence coverage %**	**Ratio**	**p-value Ratio**	**p-value Sample**	**Log 10 Ratio**
O88813	ACSL5_RAT	Long-chain-fatty-acid--CoA ligase 5	Acsl5	4	11	1.61	0.731	0.022	0.010	-0.136
Q62902	LMAN1_RAT	Protein ERGIC-53	Lman1	3	6	1.35	0.745	0.010	0.011	-0.128
P24473	GSTK1_RAT	Glutathione S-transferase kappa 1	Gstk1	2	5	5.75	0.757	0.022	0.013	-0.121
P09034	ASSY_RAT	Argininosuccinate synthase	Ass1	22	148	4.85	0.759	0.011	0.013	-0.120
P21531	RL3_RAT	60S ribosomal protein L3	Rpl3	4	6	1.74	0.764	0.019	0.014	-0.117
P50137	TKT_RAT	Transketolase	Tkt	13	42	1.61	0.767	0.040	0.014	-0.115
P01946	HBA_RAT	Hemoglobin subunit alpha-1/2	Hba1	3	43	6.34	0.773	0.000	0.019	-0.112
Q5U3Z7	Q5U3Z7_RAT	Serine hydroxymethyltransferase	Shmt2	1	4	1.59	0.773	0.022	0.017	-0.112

P04143	THRSP_RAT	Thyroid hormone-inducible hepatic protein	Thrsp	1	8	8.67	0.774	0.036	0.023	-0.112
G3V728	G3V728_RAT	4-nitrophenylphosphatase domain and non-neuronal SNAP25-like protein homolog 1	Nipsnap1	5	12	3.17	0.783	0.047	0.019	-0.107
Q4KLZ6	TKFC_RAT	Triokinase/FMN cyclase	Tkfc	11	36	3.63	0.784	0.012	0.018	-0.106
P09495	TPM4_RAT	Tropomyosin alpha-4 chain	Tpm4	2	5	3.63	0.794	0.029	0.021	-0.100
P04797	G3P_RAT	Glyceraldehyde-3-phosphate dehydrogenase	Gapdh	16	114	2.10	0.796	0.026	0.022	-0.099
P02680	FIBG_RAT	Fibrinogen gamma chain	Fgg	3	8	2.47	0.803	0.040	0.025	-0.095
Q6AXX6	F213A_RAT	Redox-regulatory protein FAM213A	Fam213a	2	6	3.49	0.819	0.006	0.031	-0.087
P63102	1433Z_RAT	14-3-3 protein zeta/delta	Ywhaz	3	14	4.90	0.825	0.021	0.043	-0.084
D3ZFQ8	D3ZFQ8_RAT	Cytochrome c-1	Cyc1	3	12	4.91	0.827	0.043	0.036	-0.083

B)

Accession	Name	Description	Gene	Peptide Count	Spectral Count	Sequence coverage %	Ratio	p-value Ratio	p-value Sample	Log 10 Ratio
P97562	ACOX2_RAT	Peroxisomal acyl-coenzyme A oxidase 2	Acox2	7	39	2.20	1.201	0.043	0.032	0.080
P41562	IDHC_RAT	Isocitrate dehydrogenase [NADP] cytoplasmic	Idh1	5	38	2.66	1.210	0.013	0.034	0.083
P17988	ST1A1_RAT	Sulfotransferase 1A1	Sult1a1	8	52	6.19	1.242	0.032	0.024	0.094
Q4V8H5	Q4V8H5_RAT	Aspartyl aminopeptidase	Dnpep	4	8	3.37	1.251	0.030	0.024	0.097
P04762	CATA_RAT	Catalase	Cat	23	123	3.61	1.258	0.016	0.021	0.100
Q9JLA3	UGGG1_RAT	UDP-glucose:glycoprotein glucosyltransferase 1	Uggt1	3	5	0.64	1.260	0.018	0.022	0.101
Q7M0E3	DEST_RAT	Destrin	Dstn	5	15	6.67	1.279	0.015	0.020	0.107
P61206	ARF3_RAT	ADP-ribosylation factor 3	Arf3	2	7	6.08	1.281	0.011	0.019	0.107
Q7TP54	FA65B_RAT	Protein FAM65B	Fam65b	3	12	1.22	1.281	0.038	0.021	0.108
Q63276	BAAT_RAT	Bile acid-CoA:amino acid N-acyltransferase	Baat	12	54	4.52	1.282	0.010	0.018	0.108
P00502	GSTA1_RAT	Glutathione S-transferase alpha-1	Gsta1	8	54	4.05	1.304	0.000	0.015	0.115
P36365	FMO1_RAT	Dimethylaniline monooxygenase [N-oxide-forming] 1	Fmo1	5	11	1.69	1.305	0.016	0.016	0.116
P08010	GSTM2_RAT	Glutathione S-transferase Mu 2	Gstm2	9	63	6.42	1.310	0.006	0.014	0.117

Q07523	HAOX2_RAT	Hydroxyacid oxidase 2	Hao2	2	5	4.82	1.325	0.016	0.011	0.122
P16303	CES1D_RAT	Carboxylesterase 1D	Ces1d	7	30	2.12	1.331	0.001	0.012	0.124
P70473	AMACR_RAT	Alpha-methylacyl-CoA racemase	Amacr	4	9	1.83	1.364	0.011	0.008	0.135
P49889	ST1E3_RAT	Estrogen sulfotransferase, isoform 3	Ste	5	11	3.39	1.387	0.000	0.008	0.142
Q5QE80	AOXC_RAT	Aldehyde oxidase 3	Aox3	3	17	1.05	1.390	0.000	0.008	0.143
P80067	CATC_RAT	Dipeptidyl peptidase 1	Ctsc	2	11	3.03	1.398	0.003	0.008	0.146

Accession	Name	Description	Gene	Peptide Count	Spectral Count	Sequence coverage %	Ratio	p-value Ratio	p-value Sample	Log 10 Ratio
Q5I0C3	MCCA_RAT	Methylcrotonoyl-CoA carboxylase subunit alpha, mitochondrial	Mccc1	2	4	1.82	1.413	0.042	0.008	0.150
Q6DGG1	ABHEB_RAT	Protein ABHD14B	Abhd14b	2	9	5.24	1.432	0.002	0.006	0.156
P62755	RS6_RAT	40S ribosomal protein S6	Rps6	3	7	6.43	1.454	0.006	0.005	0.163
Q9Z1A6	VIGLN_RAT	Vigilin	Hdlbp	3	5	1.18	1.474	0.012	0.005	0.169
P20759	IGHG1_RAT	Ig gamma-1 chain C region		3	9	2.45	1.562	0.030	0.004	0.194
D3Z8I7	D3Z8I7_RAT	Protein Gstt3	Gstt3	4	8	6.19	1.590	0.002	0.003	0.201
Q8CHM7	HACL1_RAT	2-hydroxyacyl-CoA lyase 1	Hacl1	4	6	1.55	1.625	0.002	0.003	0.211
D4ABH1	D4ABH1_RAT	Protein LOC688906	Scaf11	1	13	0.83	1.649	0.001	0.002	0.217
P50237	ST1C1_RAT	Sulfotransferase 1C1	Sult1c1	4	12	1.97	1.662	0.000	0.002	0.221
M0R4N4	M0R4N4_RAT	Protein Dhrs7l1	Dhrs7l1	1	1	2.47	1.981	0.021	0.001	0.297
P14141	CAH3_RAT	Carbonic anhydrase 3	Ca3	14	75	6.54	2.073	0.000	0.001	0.317
P63029	TCTP_RAT	Translationally-controlled tumor protein	Tpt1	1	2	7.56	2.076	0.023	0.001	0.317

Table S3. List of all dysregulated proteins in lung tissues following IONP exposure. A) Down regulated proteins and B) Upregulated proteins.Statistically significant iTRAQ ratios (P value ratio and P value sample≤0.05) for the 84 proteins that are dysregulated.

A)

Accession	Name	Description	Gene	Peptide Count	Spectral Count	Sequence coverage %	Ratio	p-value Ratio	p-value Sample	Log 10 Ratio
P01946	HBA_RAT	Hemoglobin subunit alpha-1/2	Hba1	3	43	6.34	0.375	0.000	0.000	-0.426
F1LQJ7	F1LQJ7_RAT	Protein Pck2	Pck2	1	2	1.56	0.382	0.046	0.001	-0.418
G3V9Y3	G3V9Y3_RAT	Protein Ttc25	Ttc25	1	2	2.02	0.476	0.002	0.001	-0.323
P62329	TYB4_RAT	Thymosin beta-4	Tmsb4x	2	4	29.55	0.613	0.000	0.004	-0.213
F1M6Q3	F1M6Q3_RAT	Protein Col4a2	Col4a2	1	1	0.73	0.632	0.039	0.005	-0.199
A0A0G2JSV6	A0A0G2JSV6_RAT	Globin c2	Hba-a2	3	13	0	0.657	0.024	0.007	-0.183
Q63355	MYO1C_RAT	Unconventional myosin-Ic	Myo1c	5	8	1.44	0.659	0.000	0.008	-0.181
P20070	NB5R3_RAT	NADH-cytochrome b5 reductase 3	Cyb5r3	4	10	5.65	0.666	0.037	0.013	-0.176
Q63598	PLST_RAT	Plastin-3	Pls3	4	10	1.9	0.680	0.002	0.008	-0.168
P04276	VTDB_RAT	Vitamin D-binding protein	Gc	3	9	1.47	0.703	0.009	0.014	-0.153
P21913	SDHB_RAT	Succinate dehydrogenase [ubiquinone] iron-sulfur subunit, mitochondrial	Sdhb	3	17	3.19	0.714	0.005	0.011	-0.146
P24090	FETUA_RAT	Alpha-2-HS-glycoprotein	Ahsg	3	10	2.84	0.721	0.031	0.013	-0.142

Accession	Name	Description	Gene	Peptide Count	Spectral Count	Sequence coverage %	Ratio	p-value Ratio	p-value Sample	Log 10 Ratio
P62755	RS6_RAT	40S ribosomal protein S6	Rps6	3	7	6.43	0.723	0.047	0.014	-0.141
Q63495	RAGE_RAT	Advanced glycosylation end product-specific receptor	Ager	5	25	2.24	0.725	0.001	0.014	-0.140
P47875	CSRP1_RAT	Cysteine and glycine-rich protein 1	Csrp1	1	2	8.81	0.738	0.049	0.016	-0.132
P02770	ALBU_RAT	Serum albumin	Alb	35	394	1.15	0.741	0.050	0.016	-0.130
P11762	LEG1_RAT	Galectin-1	Lgals1	5	15	7.41	0.754	0.027	0.019	-0.123

P47942	DPYL2_RAT	Dihydropyrimidinase-related protein 2	Dpysl2	3	6	1.57	0.756	0.003	0.018	-0.122
P12346	TRFE_RAT	Serotransferrin	Tf	27	145	1.72	0.759	0.007	0.020	-0.120
D3ZZZ9	D3ZZZ9_RAT	Catenin	Ctnnd1	2	5	1.39	0.764	0.022	0.022	-0.117
P02091	HBB1_RAT	Hemoglobin subunit beta-1	Hbb	3	63	6.12	0.775	0.000	0.022	-0.111
P49911	AN32A_RAT	Acidic leucine-rich nuclear phosphoprotein 32 family member A	Anp32a	2	3	3.24	0.776	0.022	0.029	-0.110
P11517	HBB2_RAT	Hemoglobin subunit beta-2		3	78	6.12	0.781	0.004	0.028	-0.107
P04639	APOA1_RAT	Apolipoprotein A-I	Apoa1	4	6	3.47	0.787	0.040	0.032	-0.104
Q9QXQ0	ACTN4_RAT	Alpha-actinin-4	Actn4	5	16	1.21	0.791	0.015	0.031	-0.102
P02680	FIBG_RAT	Fibrinogen gamma chain	Fgg	3	8	2.47	0.793	0.008	0.025	-0.101
P16617	PGK1_RAT	Phosphoglycerate kinase 1	Pgk1	4	16	4.08	0.795	0.045	0.035	-0.100
Q9Z270	VAPA_RAT	Vesicle-associated membrane protein-associated protein A	Vapa	3	6	5.62	0.803	0.029	0.036	-0.095
P14480	FIBB_RAT	Fibrinogen beta chain	Fgb	5	10	2.92	0.814	0.018	0.047	-0.089

B)

Accession	Name	Description	Gene	Peptide Count	Spectral Count	Sequence coverage %	Ratio	p-value Ratio	p-value Sample	Log 10 Ratio
P26772	CH10_RAT	10 kDa heat shock protein, mitochondrial	Hspe1	5	24	6.86	1.215	0.017	0.047	0.085
Q6AXX6	F213A_RAT	Redox-regulatory protein FAM213A	Fam213a	2	6	3.49	1.221	0.013	0.045	0.087
D3ZMX6	D3ZMX6_RAT	Protein Sntb2	Sntb2	2	5	1.72	1.223	0.030	0.047	0.087
A0A0G2K654	A0A0G2K654_RAT	Histone cluster 1 H1 family member c	Hist1h1c	1	8	0	1.225	0.020	0.043	0.088
Q4G075	ILEUA_RAT	Leukocyte elastase inhibitor A	Serpinb1a	4	6	2.64	1.231	0.050	0.044	0.090
G3V7W1	G3V7W1_RAT	Programmed cell death 6	Pdcd6	1	5	5.24	1.233	0.046	0.043	0.091
D3ZE63	D3ZE63_RAT	Protein LOC679748	LOC679748	1	8	7.83	1.265	0.014	0.036	0.102
O35244	PRDX6_RAT	Peroxiredoxin-6	Prdx6	6	26	5.36	1.267	0.050	0.037	0.103
P09606	GLNA_RAT	Glutamine synthetase	Glul	4	12	1.88	1.273	0.013	0.025	0.105
Q1HAQ0	PCAT1_RAT	Lysophosphatidylcholine acyltransferase 1	Lpcat1	1	6	1.87	1.277	0.020	0.028	0.106
P36972	APT_RAT	Adenine phosphoribosyltransferase	Aprt	2	36	7.22	1.279	0.004	0.028	0.107
P06757	ADH1_RAT	Alcohol dehydrogenase 1	Adh1	11	67	5.85	1.290	0.048	0.035	0.111

P52555	ERP29_RAT	Endoplasmic reticulum resident protein 29	Erp29	2	4	5	1.294	0.047	0.024	0.112
P50115	S10A8_RAT	Protein S100-A8	S100a8	5	35	17.98	1.305	0.009	0.025	0.116
P16232	DHI1_RAT	Corticosteroid 11-beta-dehydrogenase isozyme 1	Hsd11b1	5	38	3.82	1.326	0.011	0.020	0.123
Q9JLZ1	GLRX3_RAT	Glutaredoxin-3	Glrx3	1	3	3.86	1.329	0.040	0.022	0.123
P20673	ARLY_RAT	Argininosuccinate lyase	Asl	4	11	1.95	1.337	0.038	0.018	0.126
D4ACJ1	D4ACJ1_RAT	40S ribosomal protein S24	LOC100363469	4	13	4.51	1.339	0.006	0.018	0.127
P61206	ARF3_RAT	ADP-ribosylation factor 3	Arf3	2	7	6.08	1.346	0.012	0.020	0.129
P49889	ST1E3_RAT	Estrogen sulfotransferase, isoform3	Ste	5	11	3.39	1.356	0.015	0.017	0.132

**Accession**	**Name**	**Description**	**Gene**	**Peptide Count**	**Spectral Count**	**Sequence coverage %**	**Ratio**	**p-value Ratio**	**p-value Sample**	**Log 10 Ratio**
D3ZBN0	H15_RAT	Histone H1.5	Hist1h1b	3	5	4.95	1.357	0.025	0.023	0.132
P02466	CO1A2_RAT	Collagen alpha-2(I) chain	Col1a2	3	13	1.09	1.361	0.011	0.017	0.134
P19132	FRIH_RAT	Ferritin heavy chain	Fth1	3	8	3.85	1.361	0.000	0.016	0.134
Q63507	RL14_RAT	60S ribosomal protein L14	Rpl14	3	25	4.67	1.363	0.003	0.013	0.134
G3V8L1	G3V8L1_RAT	PYD and CARD domain containing	Pycard	2	3	4.15	1.394	0.044	0.020	0.144
P48675	DESM_RAT	Desmin	Des	2	3	1.71	1.420	0.013	0.011	0.152
D3ZQM3	D3ZQM3_RAT	Integrin alpha 3 variant B	Itga3	3	4	1.59	1.435	0.009	0.010	0.157
P68182	KAPCB_RAT	cAMP-dependent protein kinase catalytic subunit beta	Prkacb	1	3	3.42	1.443	0.035	0.009	0.159
P62836	RAP1A_RAT	Ras-related protein Rap-1A	Rap1a	3	27	6.52	1.466	0.002	0.008	0.166
P07895	SODM_RAT	Superoxide dismutase [Mn], mitochondrial	Sod2	4	23	6.31	1.489	0.005	0.007	0.173
Q3T1J1	IF5A1_RAT	Eukaryotic translation initiation factor 5A-1	Eif5a	2	4	7.79	1.498	0.002	0.007	0.176
P80067	CATC_RAT	Dipeptidyl peptidase 1	Ctsc	2	11	3.03	1.502	0.039	0.007	0.177
P06760	BGLR_RAT	Beta-glucuronidase	Gusb	1	4	1.39	1.513	0.012	0.008	0.180
P17046	LAMP2_RAT	Lysosome-associated membrane glycoprotein 2	Lamp2	1	3	2.19	1.522	0.000	0.007	0.182

P80431	COX7B_RAT	Cytochrome c oxidase subunit 7B, mitochondrial	Cox7b	1	2	10	1.524	0.046	0.007	0.183
Q5XI77	Q5XI77_RAT	Annexin	Anxa11	2	5	2.39	1.544	0.021	0.007	0.189
P02454	CO1A1_RAT	Collagen alpha-1(I) chain	Col1a1	4	20	1.24	1.549	0.000	0.006	0.190
Q7TP54	FA65B_RAT	Protein FAM65B	Fam65b	3	12	1.22	1.578	0.001	0.008	0.198
P0C170	H2A1E_RAT	Histone H2A type 1-E		1	6	5.38	1.596	0.000	0.005	0.203
P45479	PPT1_RAT	Palmitoyl-protein thioesterase 1	Ppt1	2	3	3.92	1.608	0.004	0.004	0.206

Accession	Name	Description	Gene	Peptide Count	Spectral Count	Sequence coverage %	Ratio	p-value Ratio	p-value Sample	Log 10 Ratio
P97521	MCAT_RAT	Mitochondrial carnitine/acylcarnitine carrier protein	Slc25a20	2	8	7.64	1.617	0.031	0.005	0.209
P97700	M2OM_RAT	Mitochondrial 2-oxoglutarate/malate carrier protein	Slc25a11	2	3	3.18	1.623	0.010	0.006	0.210
P09527	RAB7A_RAT	Ras-related protein Rab-7a	Rab7a	2	4	6.76	1.637	0.028	0.004	0.214
P27139	CAH2_RAT	Carbonic anhydrase 2	Ca2	4	10	6.15	1.645	0.000	0.005	0.216
P50116	S10A9_RAT	Protein S100-A9	S100a9	5	21	8.85	1.650	0.000	0.004	0.218
Q5XHY5	SYTC_RAT	Threonine--tRNA ligase, cytoplasmic	Tars	2	3	1.29	1.703	0.047	0.005	0.231
Q9Z2G8	NP1L1_RAT	Nucleosome assembly protein 1-like 1	Nap1l1	1	6	2.82	1.704	0.001	0.003	0.232
P04157	PTPRC_RAT	Receptor-type tyrosine-protein phosphatase C	Ptprc	2	3	0.86	1.710	0.005	0.004	0.233
Q9JJ54	HNRPD_RAT	Heterogeneous nuclear ribonucleoprotein D0	Hnrnpd	1	3	2.27	1.732	0.004	0.003	0.239
Q6I8Q6	Q6I8Q6_RAT	Histone H2A	Hist1h2af	1	1	5.38	1.751	0.022	0.003	0.243
Q0H8B9	CL2DB_RAT	C-type lectin domain family 2 member D11	Clec2d11	1	2	5.31	1.783	0.043	0.003	0.251
Q4FZY0	EFHD2_RAT	EF-hand domain-containing protein D2	Efhd2	2	5	2.93	1.791	0.006	0.003	0.253
Q05962	ADT1_RAT	ADP/ATP translocase 1	Slc25a4	1	5	3.02	1.939	0.000	0.002	0.288
Q9WTQ2	PODXL_RAT	Podocalyxin	Podxl	1	2	2.06	1.968	0.002	0.002	0.294
